# PTGS2-Based Network Pharmacology and Molecular Modeling Investigation of the Antioxidant Potential of Ethanolic *Allium atroviolaceum* Boiss. Extracts Across Plant Parts: Compositional Diversity, UHPLC-QTOF-IMS, and Experimental Validation

**DOI:** 10.3390/life16081256

**Published:** 2026-07-29

**Authors:** Mejdi Snoussi, Emira Noumi, Manal Mohammed Alzahrani, Khulood Fahad Alabbosh, Qusai Alsenani, Mamdouh Alshammari, Mohd Adnan, Arif Jamal Siddiqui, Riadh Ben Salah, Naourez Ktari, Karim Hosni, Vincenzo De Feo, Adel Kadri

**Affiliations:** 1 Department of Biology, College of Science, University of Ha’il, Ha’il 81451, Saudi Arabia; eb.noumi@uoh.edu.sa (E.N.); k.alabosh@uoh.edu.sa (K.F.A.); q.alsenani@uoh.edu.sa (Q.A.); mamd.alshammari@uoh.edu.sa (M.A.); mmo.adnan@uoh.edu.sa (M.A.); arif.siddiqui.uoh@gmail.com (A.J.S.); 2 Medical and Diagnostic Research Center, University of Ha’il, Ha’il 55473, Saudi Arabia; 3 Department of Chemistry, Faculty of Science, Al-Baha University, Al-Baha 65527, Saudi Arabia; 4 Laboratory of Microbial and Enzymatic Biotechnologies and Biomolecules (LBMEB), Center of Biotechnology of Sfax (CBS), Road of Sidi Mansour Km 6, Sfax 3018, Tunisia; riadh_fss@yahoo.fr (R.B.S.); naourez.ktari@yahoo.fr (N.K.); 5 Higher Institute of Biotechnology of Sfax, Soukra Road Km 4.5, Sfax 3038, Tunisia; 6 Laboratoire des Substances Naturelles, Institut National de Recherche et d’Analyse Physico-Chimique (INRAP), Biotechpole de Sidi Thabet, Ariana 2090, Tunisia; karim.hosni@inrap.rnrt.tn; 7 Department of Pharmacy, University of Salerno, Via Giovanni Paolo II, 132, 84084 Fisciano, Salerno, Italy; 8 Faculty of Science of Sfax, Department of Chemistry, Sfax University, Sfax 3000, Tunisia

**Keywords:** *Allium atroviolaceum* extracts, UHPLC-QTOF-IMS, antioxidant activity, oxidative stress, antioxidant pathway, network pharmacology, in silico approaches

## Abstract

**Background/Objectives**: *Allium atroviolaceum* Boiss. is a functional food rich in phenolic compounds; however, the contribution of different plant organs to its bioactivity remains insufficiently understood. **Methods**: An integrated approach combining UHPLC-QTOF-IMS based metabolite profiling, network pharmacology, molecular docking, and experimental antioxidant assays was employed. **Results**: Metabolite profiling revealed that the flowers were rich in anthocyanins and flavonoids; leaves in flavonol glycosides; bulbs in flavonoids, phenolics, and lipids; and flower stalks in flavonoid glycosides and sterols. Interestingly, flowers exhibited the strongest antioxidant scavenging activity, with the lowest IC_50_ values of 3.08 ± 0.21 µg/mL and 89 ± 2.8 µg/mL in the DPPH and ABTS assays, respectively. They also showed the highest FRAP response at 2 mg/mL (1.367 ± 0.010 at 2 mg/mL). Moreover, flower stalks were particularly effective in limiting lipid oxidation, displaying the greatest activity in the β-carotene bleaching assay, with an IC_50_ value of 14.75 ± 0.29 µg/mL. Network-based target prioritization highlighted several oxidative stress/inflammation-associated proteins, including EGFR, HRAS, TGFB1, BCL2, JUN, TNF, TP53, MAPK1, and PTGS2. Additionally, Caffeoyl pinoresinol showed the strongest predicted interaction with PTGS2 and was therefore selected as a candidate ligand for further computational evaluation. **Conclusions**: *A. atroviolaceum* exhibits organ-specific phytochemical diversity and potent antioxidant activity mediated through multiple molecular targets. The in silico analyses identified candidate compound–target associations related to oxidative stress and inflammatory pathways, but these findings remain predictive and require biochemical and cell-based validation.

## 1. Introduction

Oxidative stress (OS) arises when reactive oxygen species accumulate beyond the cell’s capacity to counteract them, upsetting the delicate balance that normally preserves cellular stability and function [1,2]. During normal cellular functioning, ROS are regularly produced during mitochondrial activity and other metabolic processes, and at controlled concentrations, they contribute to signaling pathways, redox homeostasis, and immune defense [3,4]. When ROS accumulate beyond the capacity of the cell’s antioxidant defenses, they can interfere with normal cellular processes, leading to damage of membrane lipids, structural and functional alterations in proteins, and changes in DNA. Together, these effects compromise cellular performance and may eventually result in cell dysfunction or death [5,6]. Antioxidant defense operates through an integrated system of enzyme-based reactions, including superoxide dismutase and catalase, together with small-molecule protectants such as glutathione and dietary antioxidants, working in concert to maintain cellular redox balance and reduce oxidative damage [7,8]. Ongoing OS has been widely associated with the development and progression of a range of chronic conditions, such as cancer, cardiovascular diseases, neurodegenerative disorders, diabetes, and persistent inflammatory diseases [9,10]. As a result, discovering bioactive compounds with potent antioxidant properties continues to be a key priority in therapeutic development. At the molecular level, OS modulates complex intracellular signaling networks that regulate cell survival, proliferation, inflammation, apoptosis, and metabolic homeostasis. Key regulatory proteins involved in these processes include EGFR (Epidermal Growth Factor Receptor), HRAS (Harvey Rat Sarcoma Viral Oncogene Homolog), TGFB1 (Transforming Growth Factor Beta 1), BCL2 (B-cell Lymphoma 2), JUN (Jun Proto-Oncogene, AP-1 Transcription Factor Subunit), ALB (Albumin), TNF (Tumor Necrosis Factor), TP53 (Tumor Protein p53), MAPK1 (Mitogen-Activated Protein Kinase 1), and PTGS2 (Prostaglandin-Endoperoxide Synthase 2), which collectively orchestrate cellular responses to oxidative stimuli [11,12]. These signaling molecules participate in interconnected pathways such as MAPK, NF-κB, and PI3K/AKT, which are critically involved in stress adaptation and inflammatory regulation [13,14]. Among them, PTGS2 (cyclooxygenase-2, COX-2) has attracted considerable attention due to its pivotal role in inflammation and OS. As an inducible enzyme that catalyzes the conversion of arachidonic acid into prostaglandins, PTGS2 mediates inflammatory responses, pain signaling, and oxidative damage [15,16]. Its overexpression is frequently associated with various OS-related pathologies, highlighting its relevance as a pharmacological target for antioxidant and anti-inflammatory agents.

Natural products have historically served as a prolific source of bioactive compounds with antioxidant potential, offering structural diversity and biological efficacy [17,18]. Medicinal plants are particularly rich in phytochemicals such as flavonoids, phenolic acids, organosulfur compounds, and terpenoids, which exhibit strong antioxidant, anti-inflammatory, and cytoprotective activities through multiple mechanisms, including ROS scavenging, metal chelation, and modulation of signaling pathways [19,20]. Within this context, *Allium atroviolaceum*, a member of the *Allium* genus, has gained increasing attention due to its diverse phytochemical composition and potential therapeutic applications [21]. Species within the *Allium* genus are well known for their high content of sulfur-containing compounds and phenolic metabolites, which contribute significantly to their antioxidant capacity and ability to modulate OS and inflammatory pathways [22,23]. Recent studies emphasize OS as a central therapeutic target in chronic diseases, where the modulation of redox homeostasis and antioxidant signaling pathways has gained increasing attention in drug discovery strategies [24,25]. Moreover, plant-derived polyphenols and secondary metabolites have demonstrated strong regulatory effects on ROS-mediated signaling and inflammatory pathways, supporting their role as multi-target antioxidant agents [26]. The integration of network pharmacology with metabolomics and molecular docking has emerged as a powerful systems biology approach for identifying bioactive compounds and elucidating their multi-target antioxidant mechanisms [27]. Despite the growing recognition of *Allium* species as important sources of bioactive phytochemicals, the organ-specific chemical composition and antioxidant potential of *Allium atroviolaceum* remain insufficiently characterized. Different plant organs often accumulate distinct classes of secondary metabolites depending on their physiological roles, developmental stage, and environmental interactions, which can lead to substantial variation in biological activity. In particular, phenolic acids, flavonoids, anthocyanins, and related phytochemicals are widely associated with antioxidant effects through direct radical scavenging, metal-chelating capacity, and reducing power. Therefore, a comparative evaluation of the bulbs, flower stalks, leaves, and flowers of *A. atroviolaceum* may provide a clearer understanding of how phytochemical distribution across organs relates to antioxidant performance. In the present study, ethanolic extracts from four organs of *A. atroviolaceum* were systematically investigated to compare their total phenolic, flavonoid, and tannin contents; characterize their metabolite profiles; and assess their in vitro antioxidant potential using DPPH radical scavenging, β-carotene bleaching, and ferric-reducing antioxidant power assays. Because antioxidant phytochemicals may also interact with molecular pathways associated with oxidative stress and inflammation, an additional exploratory in silico workflow was employed to prioritize candidate bioactive compounds and putative protein targets related to these processes. For this purpose, predicted targets of selected phytochemicals were intersected with oxidative stress-associated genes, followed by network analysis and molecular modeling to identify candidate compound–target interactions of potential biological relevance.

Accordingly, the objectives of this study were to: (i) compare the phytochemical composition of bulbs, flower stalks, leaves, and flowers of *A. atroviolaceum*; (ii) evaluate the antioxidant activity of their ethanolic extracts using complementary in vitro assays; and (iii) explore, through network pharmacology and molecular modeling, putative oxidative stress/inflammation-related targets associated with selected phytochemicals. The computational findings are intended to provide a hypothesis-generating framework for future mechanistic studies rather than direct experimental confirmation of molecular antioxidant mechanisms.

## 2. Results and Discussion

This work provides a novel integrated investigation of *Allium atroviolaceum* by combining high-resolution mass spectrometry-based metabolomic profiling across four plant organs (bulbs, flower stalks, leaves, and flowers) with network pharmacology and in silico analyses. Its originality lies in the unprecedented multi-organ coverage coupled with a systems-level strategy that links chemical diversity to biological function. This study uniquely integrates UHPLC-QTOF-IMS profiling with molecular modeling and target prediction, followed by KEGG pathway mapping highlighting apoptosis, cell cycle regulation, and cellular stress responses. To our knowledge, this is the first work of this type to connect such a comprehensive metabolomic resolution with computational mechanistic insights and experimental antioxidant validation. Overall, this multi-layered framework establishes a distinctive and original approach for elucidating the antioxidant potential of *Allium atroviolaceum*.

### 2.1. Total Phenolic, Flavonoid, and Tannin Contents of Bulbs, Flower Stalks, Leaves, and Flowers

The phytochemical analysis (Table 1) revealed clear organ-specific differences in the distribution of phenolic, flavonoid, and tannin contents in EAAE. Flowers consistently exhibited the highest levels of phenolics, flavonoids, and tannins, indicating a marked enrichment compared to the other plant organs. In contrast, flower stalks, leaves, and bulbs showed lower and variable contents depending on the compound class, reflecting differential metabolic allocation among tissues. Our results corroborate those reported by Emir and Emir [28], who confirmed that both TPC and TFC in *A. atroviolaceum* from two sites varied among plant organs. In Kemalpaşa (İzmir), the flower exhibited the highest TPC (33.72 ± 1.4 mg GAE/g extract) and TFC (8.63 ± 1.1 mg QE/g extract), followed by the bulb, while the stem showed the lowest values, indicating the trend: flower, bulb, stem. In Tire (İzmir), the flower also showed the highest TPC (25.81 ± 1.8 mg GAE/g extract), whereas the bulb exhibited the highest TFC (9.17 ± 0.8 mg QE/g extract), followed by the stem and then the flower.

### 2.2. Metabolite Profiling of AAE Across Bulbs, Flower Stalks, Leaves, and Flowers Using UHPLC-QTOF-IMS

UHPLC-QTOF-IMS-based metabolite profiling (Table 2) and chromatograms (Supplementary Figure S1 (Figure S1A–C)) demonstrated clear differences in the chemical composition among the four organs (bulbs, flower stalks, leaves, and flowers), highlighting an organ-specific distribution of secondary metabolites. Overall, the ethanolic extract from bulbs was dominated by Caffeoyl-6-O-hexoside dihydrate (31.52%), Naringin (18.46%), 3,4-Dicaffeoylquinic acid (13.13%), Liquiritin apioside (11.51%), and 9-Hydroperoxyoctadecadienoic acid (8.56%), while the extract of flowers stalks contain mainly β-sitosterol (26.87%), Caffeoyl-6-O-hexoside dihydrate (22.55%), Peonidin 3-glucoside (10.55%), Hydroxygenkwanin (9.30%), Hyperoside (4.52%), and Cyanidin 3-O-glucoside (4.29%). The ethanolic extract was particularly rich in Hyperoside (36.60%), Cyanidin 3-O-glucoside (14.44%), Isorhamnetin (2.92%), Peonidin 3-glucoside (2.46%), and Hydroxygenkwanin (1.21%), while flower ethanolic extract contained Apigenin (18.85%), Isorhamnetin (14.19%), Cyanidin (12.75%), Cyanidin 3-O-glucoside (6.62%), and Hydroxygenkwanin (5.40%). MS/MS fragmentation spectra of the principal metabolites identified by the UHPLC-QTOF-IMS technique are reported in Supplementary Figure S2.

These findings, obtained through high-resolution metabolite profiling, indicate a distinct organ-dependent metabolic specialization within the plant. The chemical composition of *A. atroviolaceum* leaves includes a wide range of volatile compounds identified from different extraction methods. These comprise sulfur-containing compounds such as dimethyl trisulfide, ethyl linolenate, phytol, methyl 1-propenyl disulfide, diallyl disulfide, and various dithiane and trithiane derivatives, along with oxygenated constituents like ethyl linolenate and phytol. Other identified components include hydrocarbons and terpenes such as limonene and isocitronellene, as well as ketones, aldehydes, and fatty acid derivatives including tetradecanoic acid and 6,10,14-trimethylpentadecan-2-one. Overall, the leaves are characterized by a chemically diverse profile dominated by organosulfur and volatile secondary metabolites [29,30]. Using LC-ESI-MS/MS analysis, it has been reported that the phytochemical profile of *A. atroviolaceum* showed clear variation among its organs collected from two localities (Kemalpaşa and Tire) [28]. The bulbs were mainly characterized by high levels of gallic acid in Kemalpaşa and naringenin in Tire, along with other flavonoids such as kaempferol and genistein. The flowers were dominated by phenolic acids, particularly hydroxybenzoic acid derivatives in Kemalpaşa, while Tire showed higher levels of flavonoids such as Isorhamnetin and genistein. The leaves (aerial parts) were mainly enriched with phenolic acid derivatives such as ferulic and *p*-coumaric acids, together with flavonoids including luteolin, quercetin, and kaempferol derivatives [28].

### 2.3. Antioxidant Activity of EAAE from Bulbs, Flower Stalks, Leaves, and Flowers

**Figure 1:**
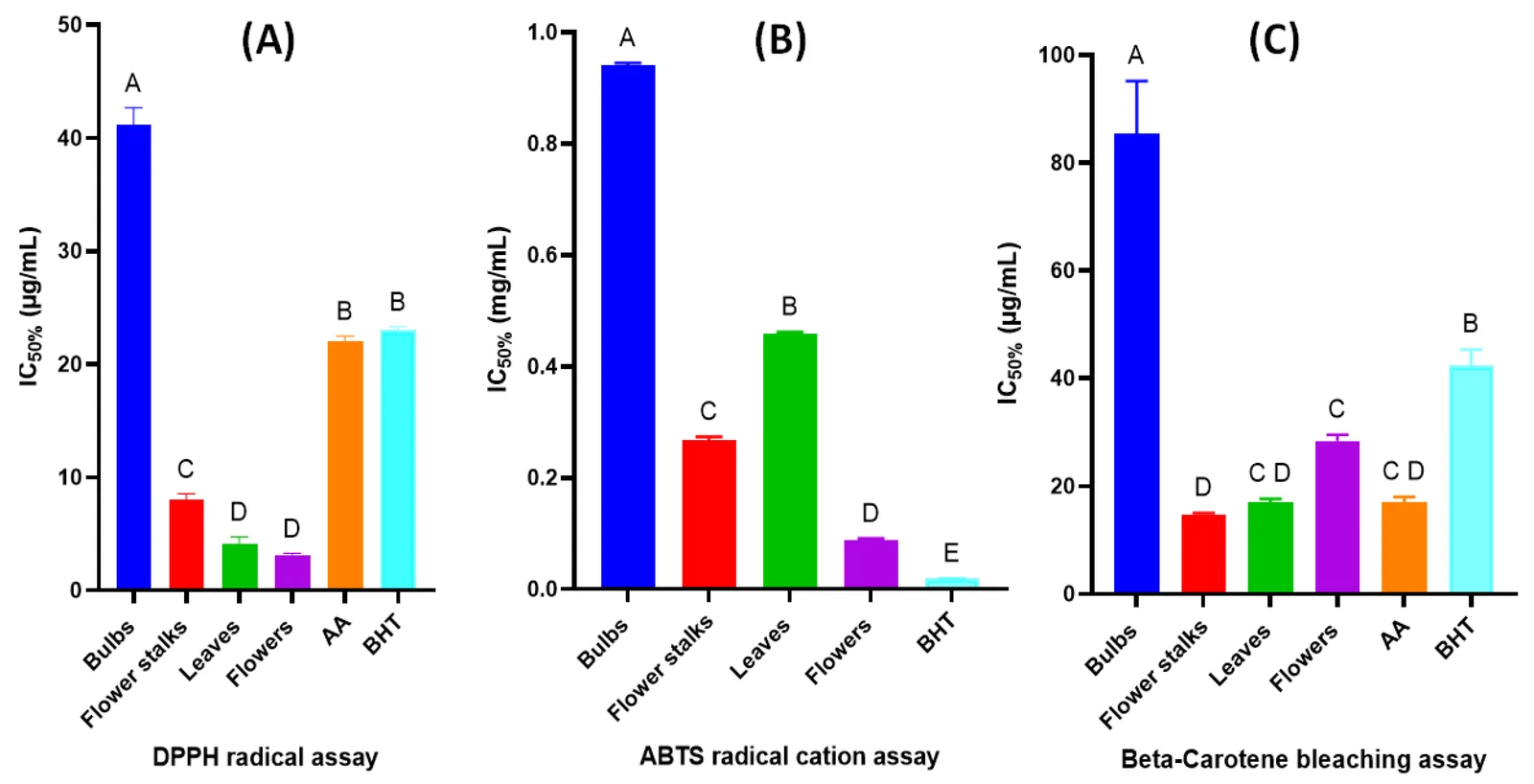
Antioxidant activities of EAAE via (**A**) DPPH (2,2-Diphenyl-1-picrylhydrazyl Radical Scavenging Assay), (**B**) ABTS (2,2′-Azino-bis(3-ethylbenzothiazoline-6-sulfonic acid) Radical Cation Decolorization Assay), and (**C**) *β*-carotene assays. Data are presented as mean ± SD from three independent experiments. Statistical significance was assessed using two-way ANOVA followed by Tukey’s post hoc test. Different letters (A to E) in the compact letter display indicate significant differences (*p* < 0.05).

In the DPPH radical scavenging assay (Figure 1A), the samples exhibited a clear variation in antioxidant capacity, as indicated by their IC_50_ values. Flowers demonstrated the most potent activity (3.08 ± 0.205 µg/mL), which is about seven-times higher than both standards, closely followed by leaves (4.15 ± 0.583 µg/mL) and flower stalks (7.99 ± 0.561 µg/mL), reflecting strong free radical neutralization potential. In contrast, bulbs demonstrated significantly reduced activity (41.22 ± 1.46 µg/mL). The reference standards displayed moderate antioxidant capacity, with IC_50_ values of 22.0 ± 0.50 and 23.0 ± 0.30 µg/mL, respectively, for ascorbic acid and BHT. Overall, the lower IC_50_ values of leaves and flowers indicate superior hydrogen-donating ability and higher radical scavenging efficiency.

For the ABTS radical cation assay (Figure 1B), the IC_50_ values of the tested samples show clear differences in inhibitory potency. Flowers were found to possess the highest scavenging activity (89 ± 2.8 µg/mL), followed by flower stalks (268 ± 9.2 µg/mL), leaves (458 ± 5.7 µg/mL), and bulbs (940 ± 7.1 µg/mL), indicating stronger inhibition at lower concentrations. The reference antioxidant BHT showed the highest potency with an IC_50_ of 18.8 ± 0.85 µg/mL. Low standard deviations suggest the results are reliable and reproducible. Overall, BHT is the most effective inhibitor, while BE4 is the most promising among the extracts.

In the *β*-carotene bleaching assay (Figure 1C), which evaluates the inhibition of lipid peroxidation, flower stalks showed potent activity (14.75 ± 0.291 µg/mL), followed by flowers (16.91 ± 0.723 µg/mL); however, leaves exhibited moderate effectiveness (28.41 ± 1.11 µg/mL) and bulbs displayed the lowest antioxidant activity once (85.42 ± 9.75 µg/mL). The standards showed intermediate inhibition capacities, with IC_50_ values of 17.0 ± 1.0 and 42.0 ± 3.5 µg/mL for ascorbic acid and BHT. These findings suggest that, while flowers and leaves excel in radical scavenging, flower stalks may be more effective in preventing lipid oxidation.

**Figure 2:**
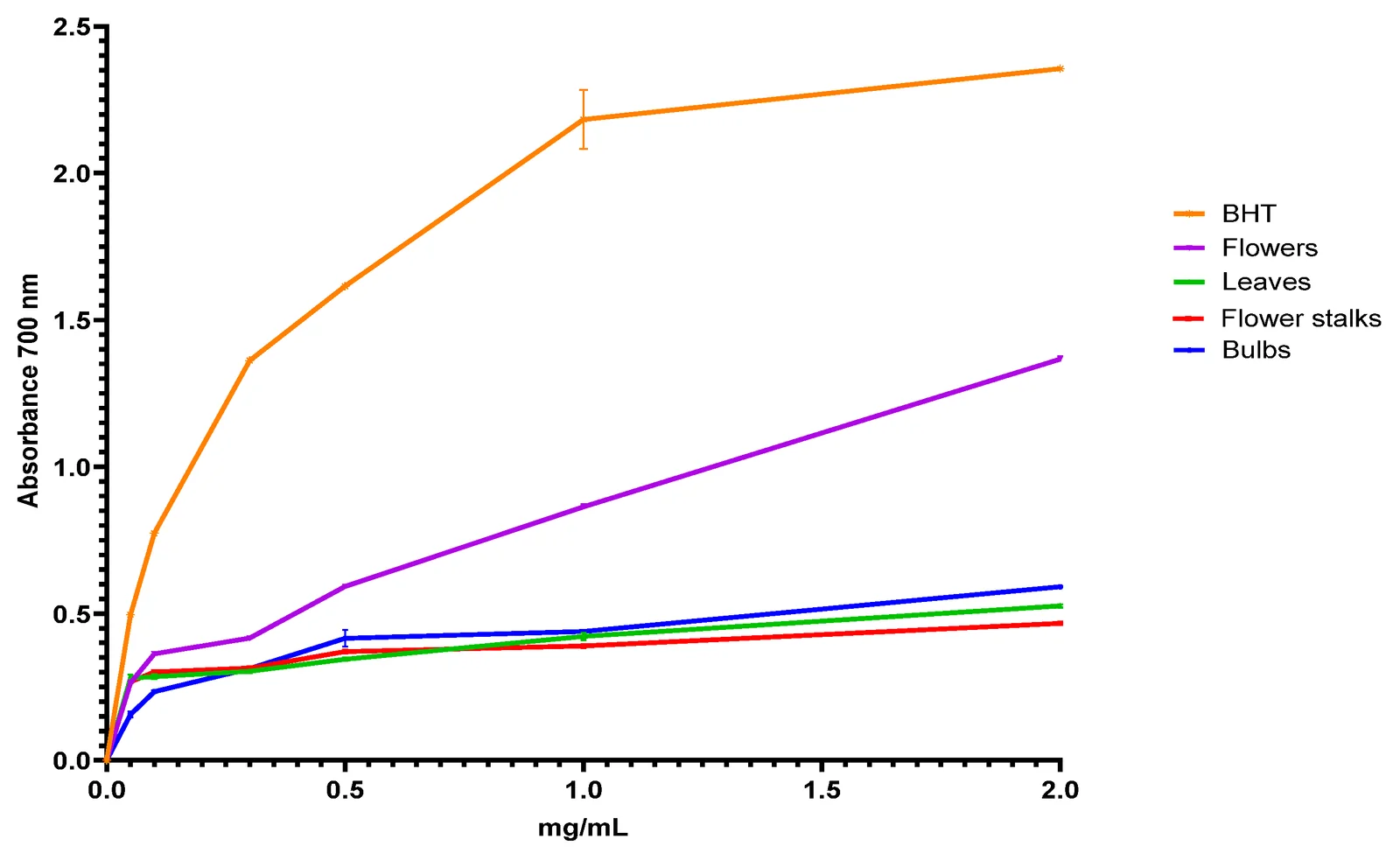
Reducing power of EAAE from bulbs, flower stalks, leaves, and flowers via FRAP assay as compared to BHT (Butylated Hydroxytoluene).

The FRAP results (Figure 2) showed a concentration-dependent increase in absorbance for all extracts, confirming their antioxidant-reducing capacity. At 2 mg/mL, flowers exhibited the highest activity (1.367 ± 0.010), followed by bulbs (0.592 ± 0.001), leaves (0.527 ± 0.004), and flower stalks (0.468 ± 0.005).

A comparative evaluation of antioxidant activity showed clear differences between the two localities of *A. atroviolaceum* [28]. For DPPH scavenging activity, the strongest effect was observed in Kemalpaşa flowers (IC_50_ = 42.66 µg/mL), followed by Kemalpaşa bulbs (51.93 µg/mL), while the weakest activity was recorded in Tire flowers (62.44 µg/mL) and leaves (97.58 µg/mL). For CUPRAC-reducing capacity, the highest value was also in Kemalpaşa flowers (182.92 mg TE/g extract), followed by Tire bulbs (131.77 mg TE/g extract) and Kemalpaşa bulbs (124.81 mg TE/g extract), whereas the lowest activity was observed in Tire flowers (109.25 mg TE/g extract) and leaves (68.44 mg TE/g extract).

### 2.4. Network Pharmacology

**Figure 3:**
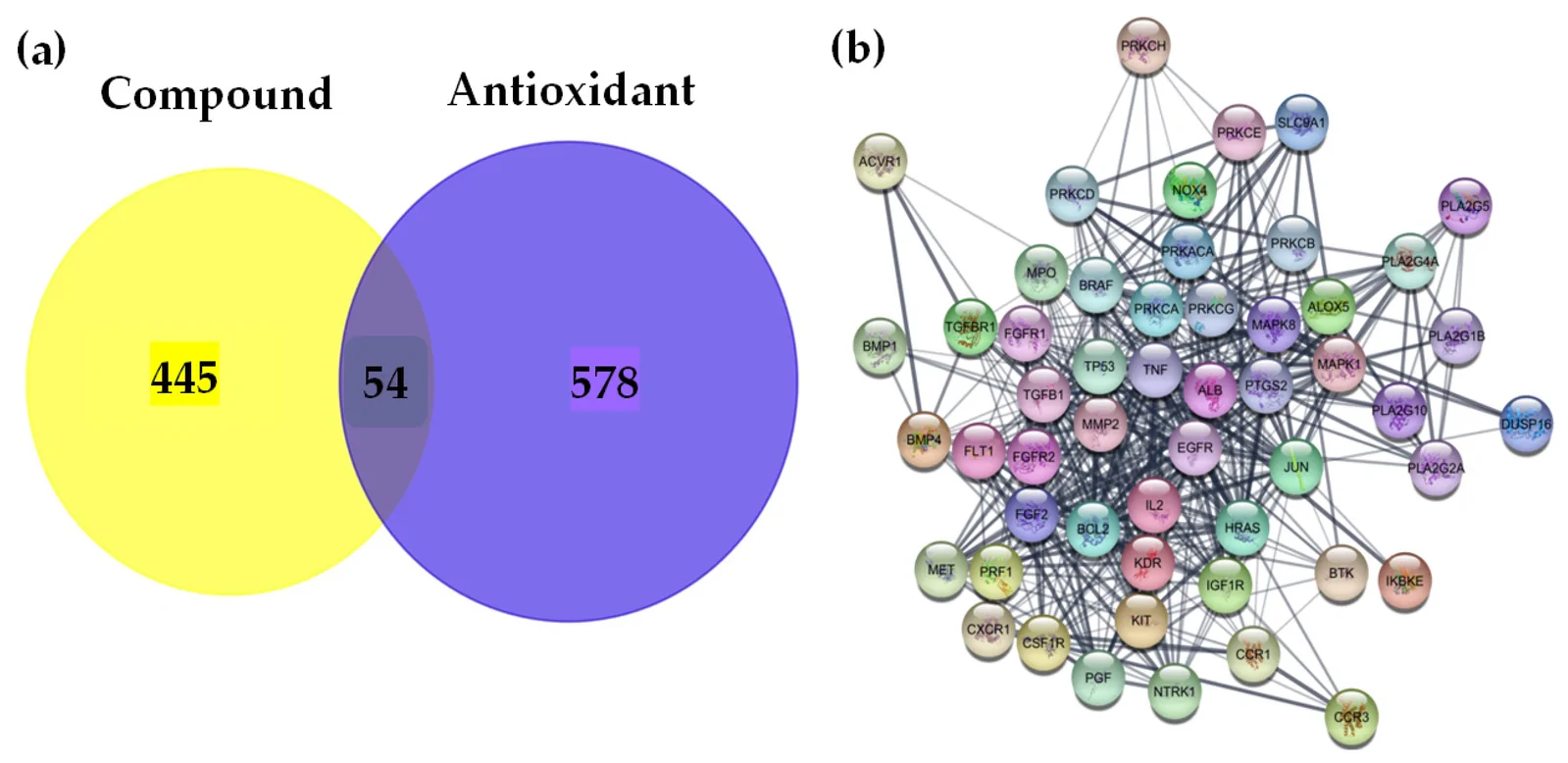
(**a**) SwissTargetPrediction identified 445 putative targets for the selected *A. atroviolaceum* phytochemicals, and overlap analysis with 578 oxidative stress-associated genes retrieved from GeneCards, MalaCards, and OMIM revealed 54 shared targets. (**b**) A common protein–protein network comprising genes from phytochemical targets and disease targets.

To complement the phytochemical profiling and in vitro antioxidant assays, a network pharmacology workflow was applied as an exploratory strategy to prioritize candidate molecular targets potentially associated with oxidative stress- and inflammation-related processes. In fact, 445 putative targets of the selected phytochemicals identified in *A. atroviolaceum* extracts were predicted using SwissTargetPrediction, while 578 oxidative stress-associated genes were compiled from GeneCards, MalaCards, and OMIM. Intersection of these datasets yielded 54 overlapping genes, which were visualized in a Venn diagram and subsequently used to construct a protein–protein interaction network (Figure 3a,b).

**Figure 4:**
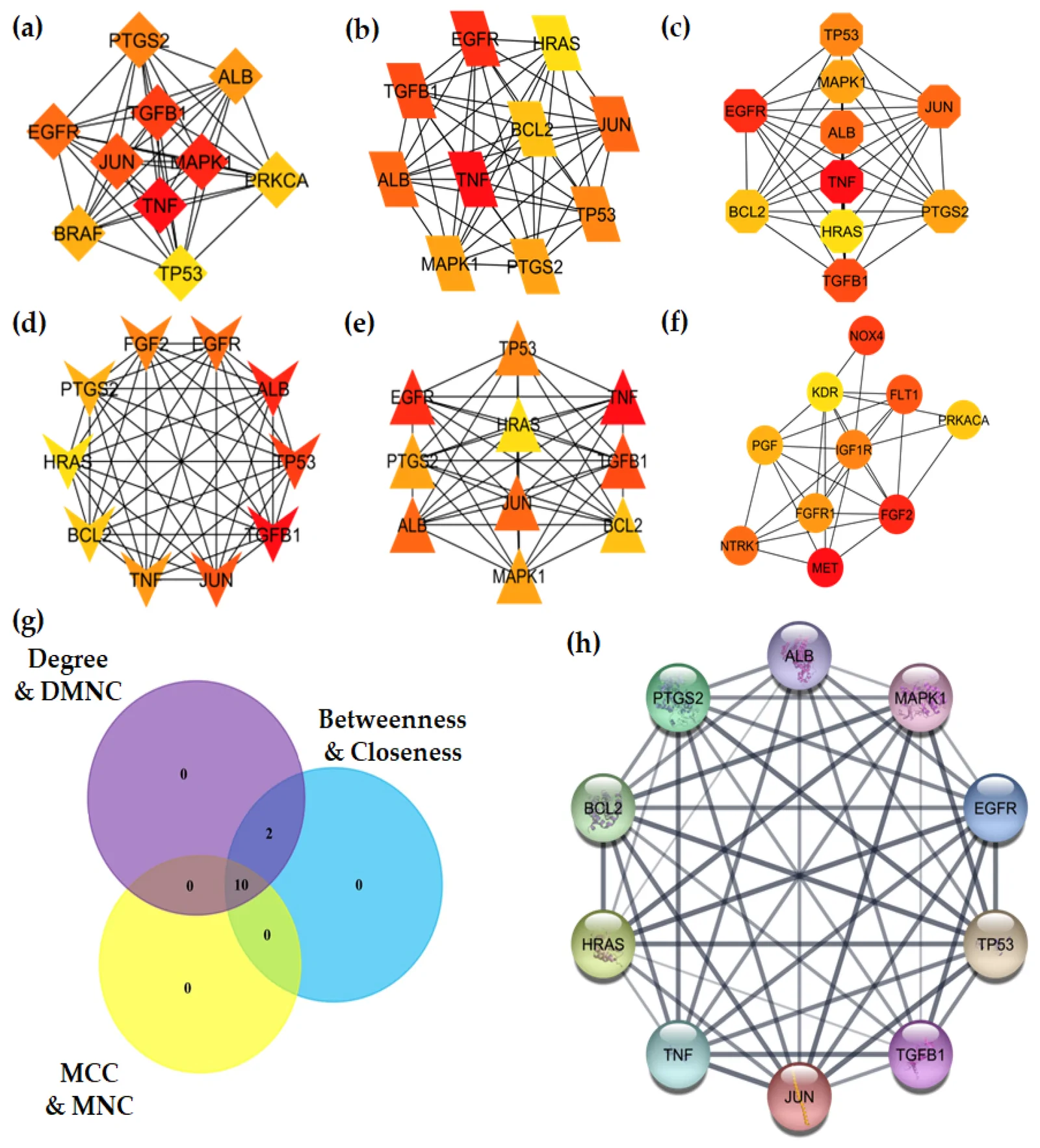
Top hub genes based on degrees of (**a**) betweenness, (**b**) closeness, (**c**) centrality, (**d**) MCC (maximal clique centrality), (**e**) MNC (maximum neighborhood component (MNC), and (**f**) DMNC (density of maximum neighborhood component); (**g**) Venn diagram of common genes of all the parameters; (**h**) common genes from all 6 parameters.

A total of 54 overlapping genes were obtained and subjected to network topological analysis, including degree centrality, closeness centrality, betweenness centrality, maximal clique centrality (MCC), maximum neighborhood component (MNC), and density of maximum neighborhood component (DMNC) analyses to identify the most significant hub targets. Based on these parameters, ten key hub genes—EGFR, HRAS, TGFB1, BCL2, JUN, ALB, TNF, TP53, MAPK1, and PTGS2—were identified and selected for further analysis (Figure 4). These genes are broadly associated with inflammatory signaling, apoptosis, cellular stress responses, and redox-related regulatory pathways, and therefore represent plausible candidates for further investigation in the context of antioxidant and cytoprotective phytochemicals.

Biological interpretation of the prioritized network suggested that several of these hub genes (Figure 4) are closely involved in oxidative stress-related pathways through their roles in regulating inflammatory signaling, cellular stress responses, and apoptosis. Proteins such as TNF, TGFB1, and JUN promote inflammatory signaling and ROS production, while MAPK1 and HRAS regulate stress-response pathways and cell survival [31,32]. TP53 and BCL2 control apoptosis under oxidative conditions, and EGFR signaling contributes to ROS-mediated cellular responses [33,34]. Among these targets, PTGS2 (cyclooxygenase-2) plays a critical role in oxidative and inflammatory pathways by catalyzing the conversion of arachidonic acid into prostaglandins, which promote inflammation and oxidative damage [35]. However, because these genes were identified through target prediction and network topology rather than direct biochemical or cellular assays, they should be interpreted as putative network-level candidates rather than experimentally confirmed mediators of the antioxidant effects observed in the chemical assays.

**Figure 5:**
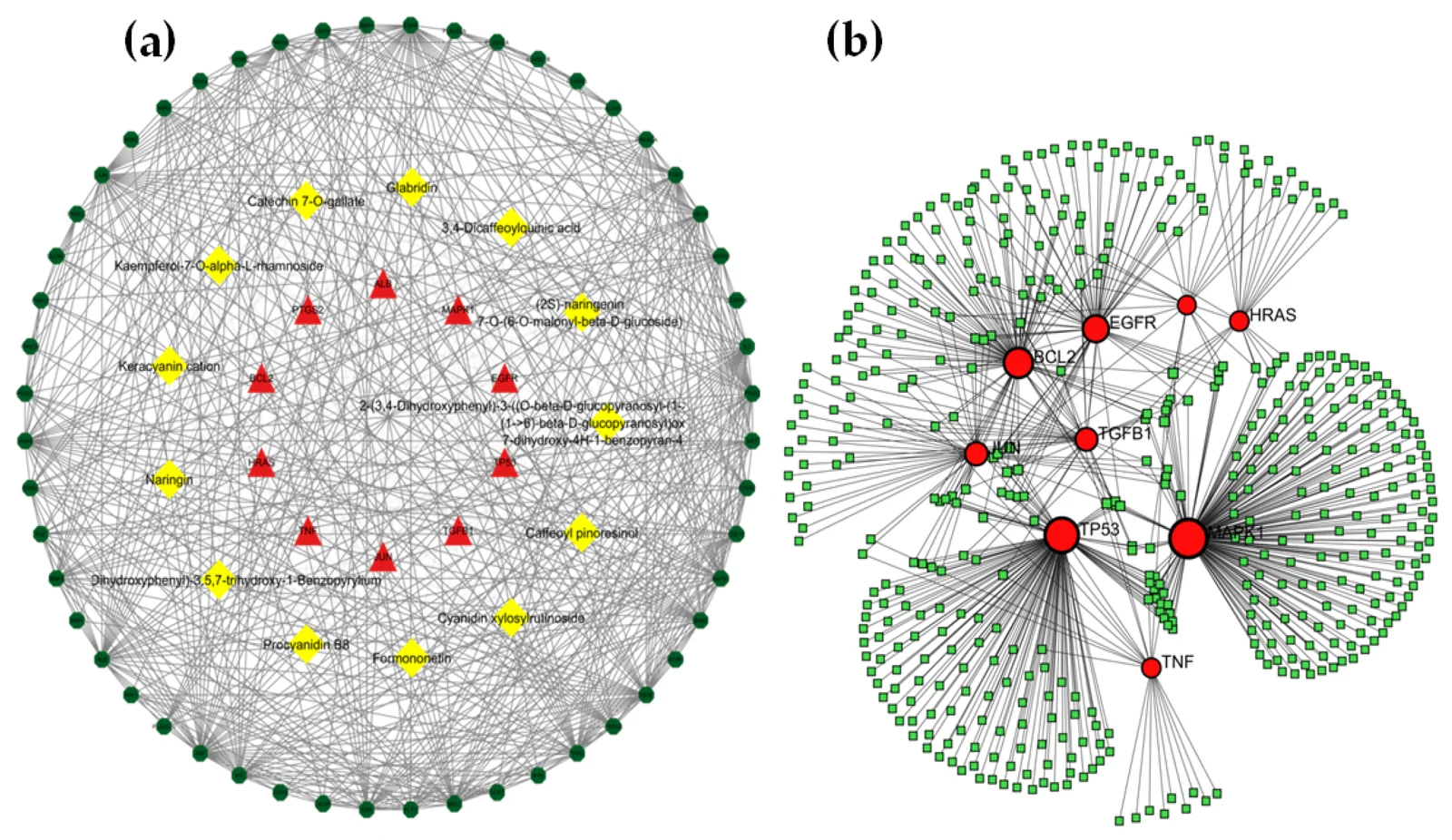
(**a**) Compound–target interaction network. (**b**) Gene–TF–mRNA network.

To better understand the molecular mechanisms underlying the antioxidant potential of *A. atroviolaceum*, interaction networks were constructed to examine relationships among hub genes, phytochemicals, and regulatory miRNAs [36]. A compound–target network using selected bioactive phytochemicals, including catechin derivatives, glabridin, and kaempferol glycosides, revealed multi-target interactions with key hub genes (Figure 5a), reflecting the multi-target nature of natural compounds. Integration with miRNA data (Figure 5b) showed extensive connections between hub genes and regulatory miRNAs, indicating additional post-transcriptional control. These analyses were included to provide a systems-level overview of possible phytochemical–target relationships rather than to infer direct target modulation by the extracts.

**Figure 6:**
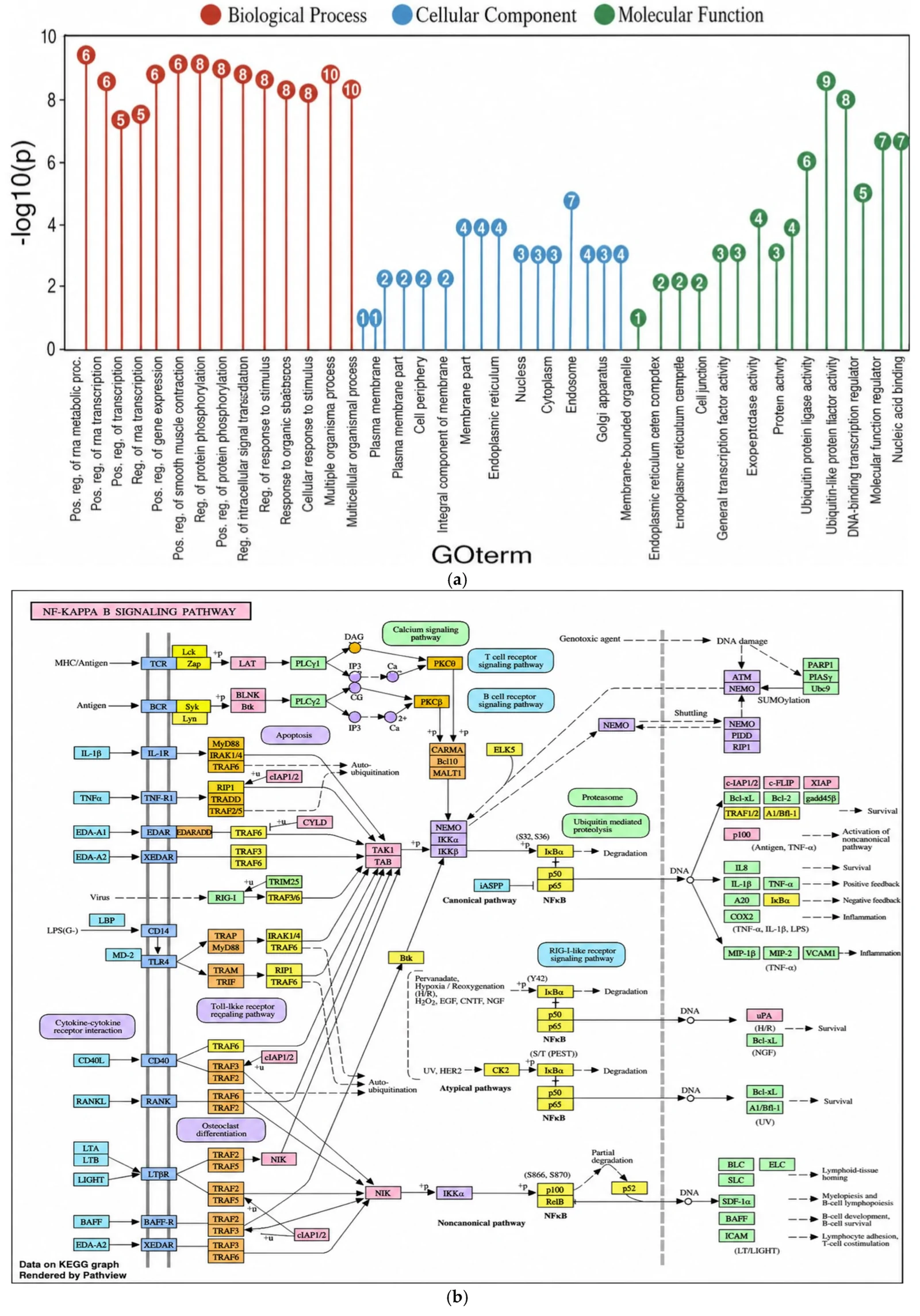

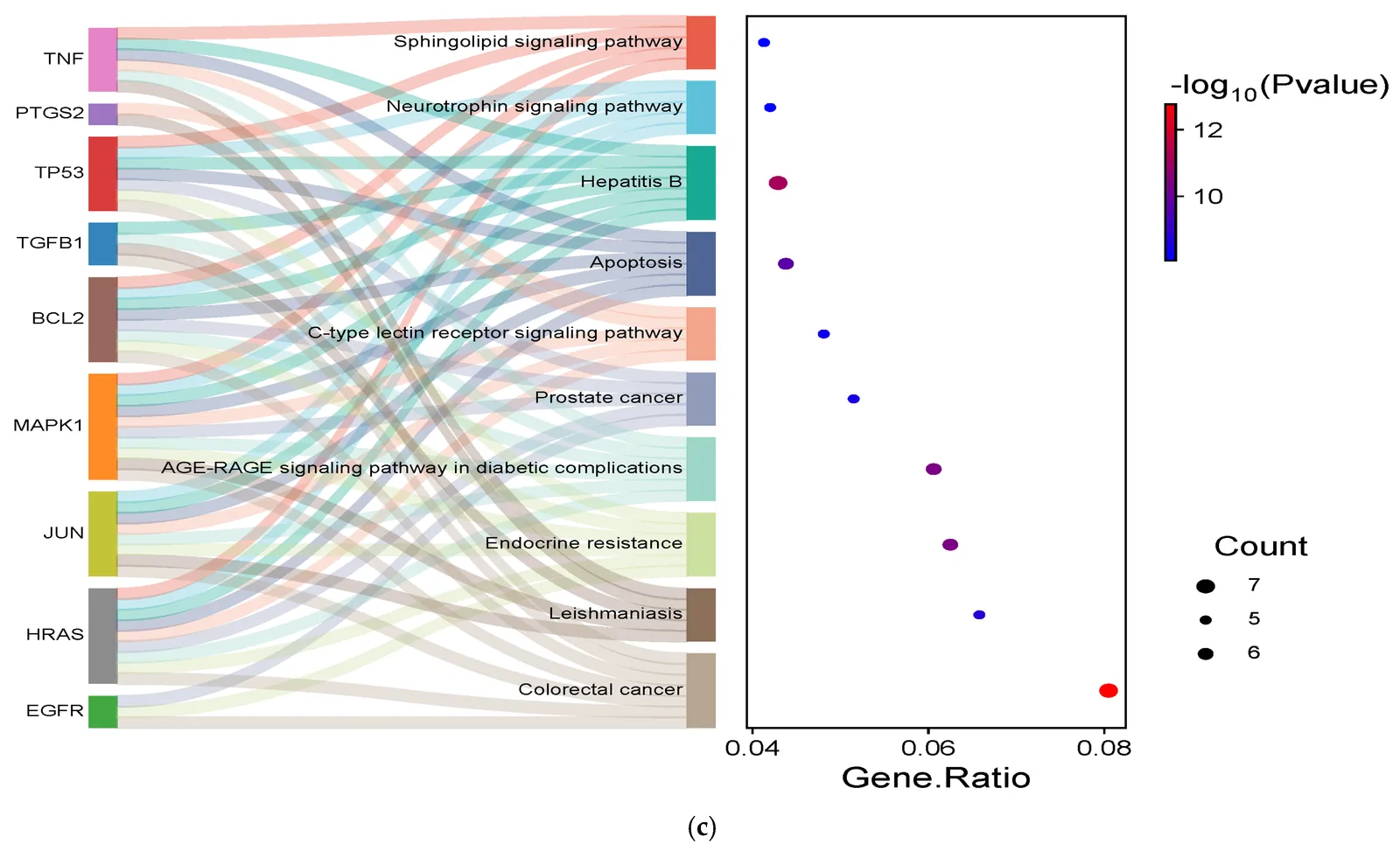
Functional and pathway enrichment of hub genes: (**a**) GO enrichment of biological process, cellular component, and molecular function; (**b**) KEGG pathways highlighting apoptosis, cell cycle, and stress responses; (**c**) Sankey diagram showing genepathway interactions.

Functional enrichment analysis of the prioritized genes further supported their association with stress-responsive and inflammation-related pathways. Gene Ontology (Figure 6a) highlighted biological processes such as apoptotic signaling, transcriptional regulation, and stress responses, with molecular function terms emphasizing binding and catalytic activities, and cellular component analysis localizing proteins mainly to the nucleus and organelles. Collectively, these results underscore the central roles of the identified genes in cellular regulation and oxidative stress pathways.

Pathway mapping through the KEGG database (Figure 6b) contextualized the roles of these genes within complex signaling networks [37]. Several genes converge on pathways related to apoptosis, cell cycle regulation, and oxidative stress responses, suggesting coordinated participation in essential cellular processes. The network complexity indicates that these genes may act as hub nodes, integrating multiple upstream and downstream signals, consistent with their potential as regulatory or therapeutic targets.

Integration of gene–pathway interactions using a Sankey diagram (Figure 6c) highlighted the pleiotropic roles of key genes such as TNF, TP53, and BCL2, which contribute to apoptotic signaling, cancer-related pathways, and inflammatory responses. The size and significance of each connection reflect the relative influence of individual genes, emphasizing their prioritization for experimental validation. Together, these findings indicate that the phytochemicals detected in *A. atroviolaceum* may be linked, at a predictive systems level, to multiple oxidative stress- and inflammation-related signaling nodes.

Among the prioritized hub genes, PTGS2 was selected as a representative target for subsequent docking analysis because it emerged consistently in the topological screening and has recognized biological relevance to inflammatory and oxidative stress-associated signaling. Nevertheless, this selection should be interpreted as a target-prioritization step within an exploratory computational workflow, rather than as evidence that PTGS2 is the validated molecular mediator of the antioxidant activity observed for *A. atroviolaceum* extracts. Since no enzymatic PTGS2/COX-2 inhibition assay or cell-based inflammatory/oxidative stress model was included in the present study, the network pharmacology findings are best regarded as hypothesis-generating results that can guide future mechanistic validation.

### 2.5. Molecular Docking and Dynamic Simulations Analyses

In silico techniques, including molecular docking, molecular dynamics simulations, density functional theory (DFT), and ADMET/drug-likeness evaluation, provide comprehensive insights into ligand–protein interactions by predicting binding, assessing stability and flexibility, analyzing electronic properties, and evaluating pharmacokinetic and toxicity profiles [38,39,40,41,42].

**Figure 7:**
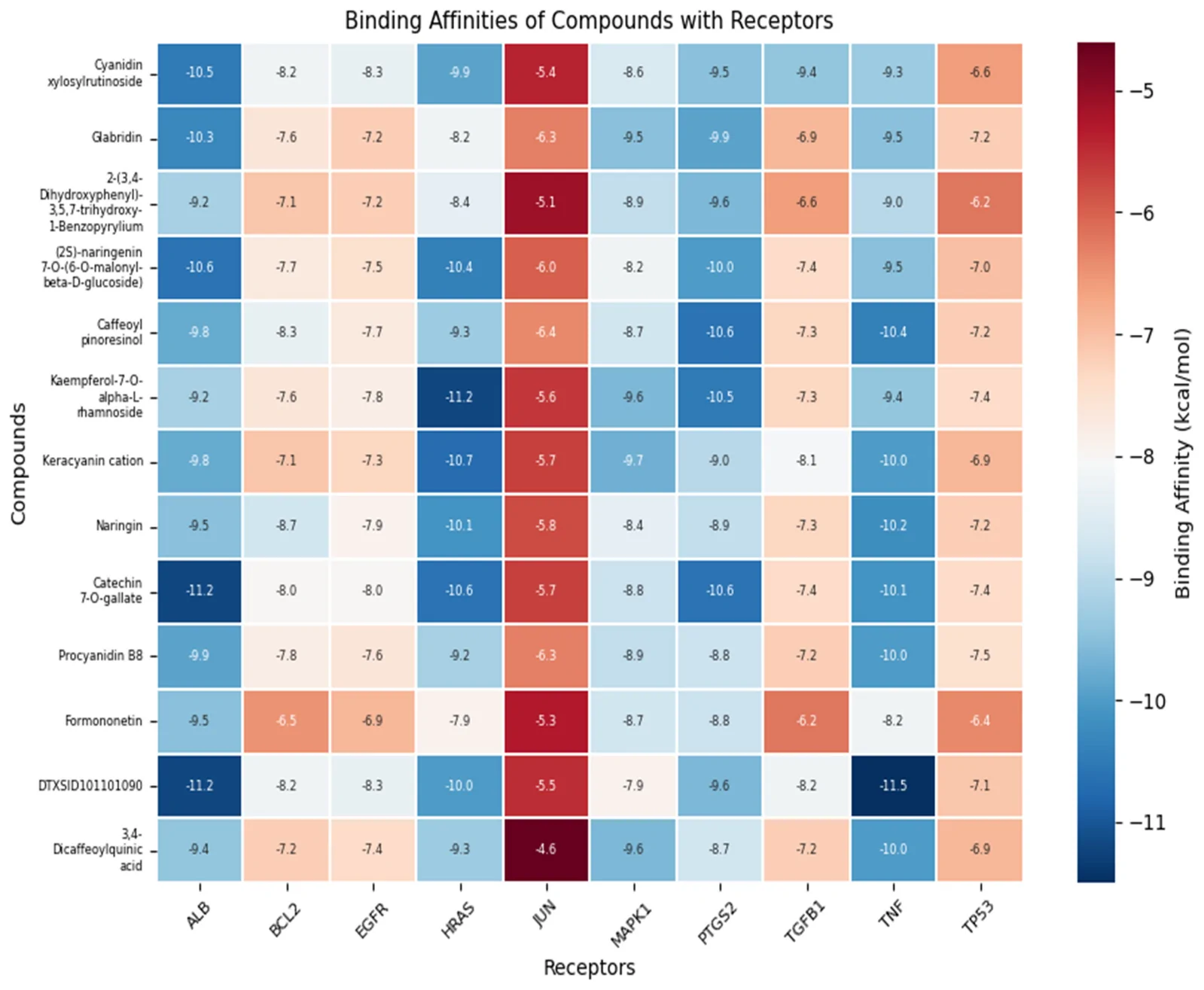
Molecular docking heatmap of selected phytochemicals against hub genes.

Molecular docking assessed interactions between phytochemicals from *A. atroviolaceum* (Figure 7) and key protein targets in oxidative stress pathways [43]. Docking predicts ligand orientation within protein active sites and estimates interaction strength, with more negative binding energies (kcal/mol) indicating stronger, more stable complexes [44]. This identifies lead compounds with optimal binding and provides insights into potential antioxidant effects.

The heatmap shows binding affinities of 13 phytochemicals against hub targets, ranging from −4.6 to −11.5 kcal/mol. Tumor necrosis factor, HRas, and PTGS2 display strong binding with several ligands, indicating favorable binding pockets. In contrast, c-Jun shows weak binding across all compounds, suggesting poor ligand compatibility.

**Figure 8:**
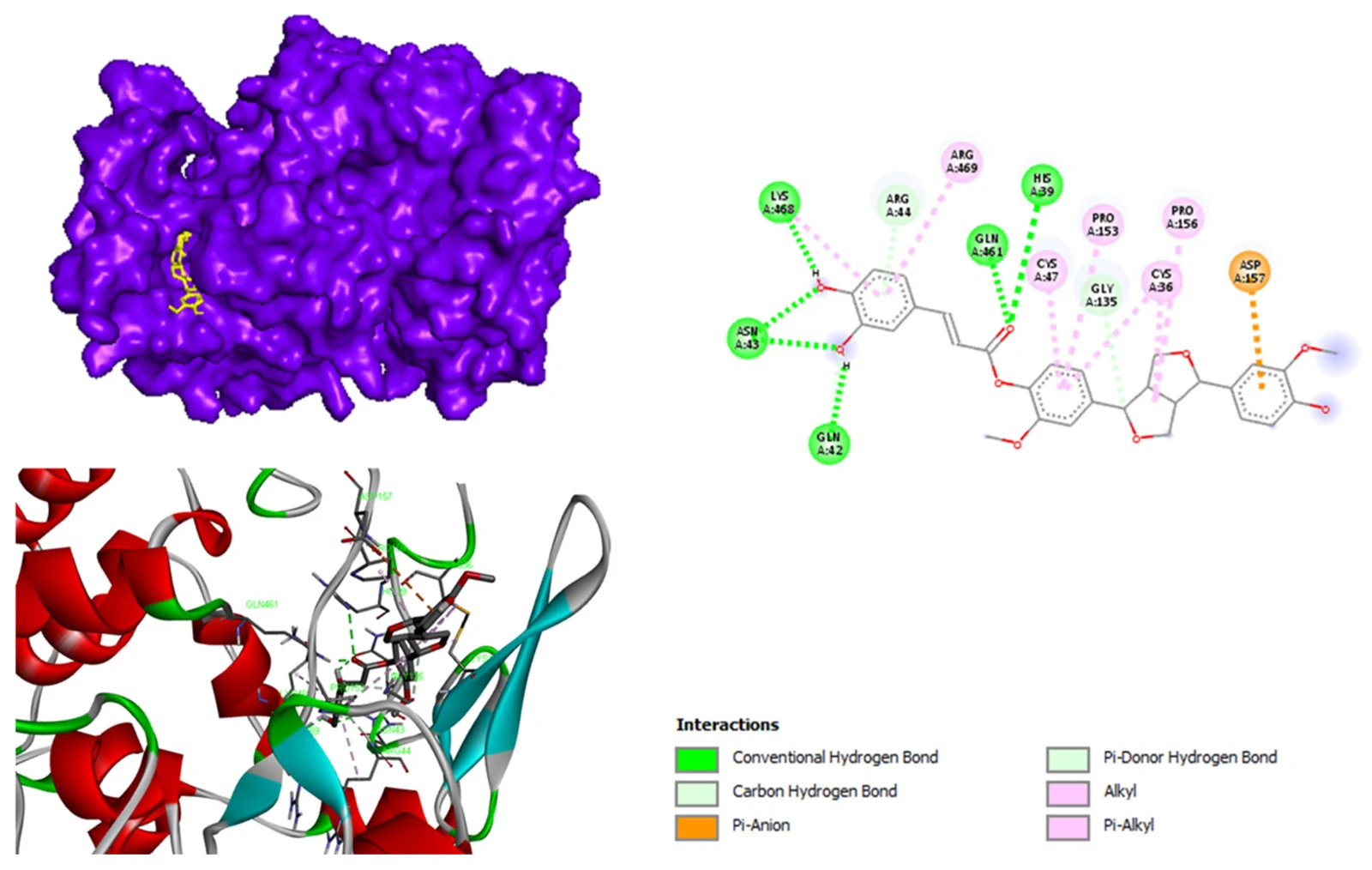
The 2D and 3D interactions of PTGS2 with Caffeoyl pinoresinol.

In PTGS2, several compounds bind below −9.0 kcal/mol, with Caffeoyl pinoresinol showing the strongest interaction at −10.6 kcal/mol, reflecting high stability and structural complementarity. Its phenolic hydroxyl groups and conjugated aromatic systems enable stabilizing hydrogen bonds and π–π interactions. Other compounds, such as catechin derivatives and kaempferol glycosides, also bind PTGS2, but Caffeoyl pinoresinol stands out for its consistent, selective interaction. Detailed inspection of the docked PTGS2–Caffeoyl pinoresinol complex showed that the ligand was accommodated within the active-site region and formed multiple non-covalent interactions with surrounding residues (Figure 8). Hydrogen bonds were observed with HIS39, GLN42, CYS47, CYS36, and THR41, while additional hydrophobic and van der Waals contacts contributed to the stabilization of the docked pose. The PTGS2–catechin pentoside complex also showed extensive hydrogen bonding, including interactions with CYS47, GLN461, HIS39, CYS36, THR41, and CYS41, whereas glabridin interacted through hydrogen bonding with CYS36 and GLN42, accompanied by hydrophobic contacts. Notably, these interaction profiles overlapped with residues reported to participate in the binding of established PTGS2 inhibitors such as celecoxib, etoricoxib, and rofecoxib, suggesting that the prioritized phytochemicals occupy a similar active-site region. However, these computational observations indicate potential binding compatibility rather than equivalent inhibitory activity and require experimental validation. Unlike ligands with broad, non-specific binding, its selectivity supports further investigation as a targeted antioxidant and anti-inflammatory agent.

From a biological perspective, targeting PTGS2 is highly relevant for antioxidant activity because this enzyme catalyzes the formation of pro-inflammatory prostaglandins that contribute to oxidative stress. Therefore, strong inhibition of PTGS2 directly correlates with potential antioxidant and anti-inflammatory effects. The strong binding affinity of Caffeoyl pinoresinol to PTGS2 suggests that it may effectively suppress this pathway. The 2d and 3d interactions of PTGS2 with Caffeoyl pinoresinol are shown in Figure 8.

Accordingly, the PTGS2–Caffeoyl pinoresinol interaction should be regarded as a computationally prioritized ligand–target pair that may warrant future biochemical evaluation, rather than as a validated explanation for the antioxidant activity observed in A. atroviolaceum extracts. In the absence of direct PTGS2/COX-2 inhibition assays or cell-based inflammatory/oxidative stress experiments, the docking results are best interpreted as a comparative molecular modeling exercise that helps identify plausible phytochemical candidates for follow-up mechanistic studies.

**Figure 9:**
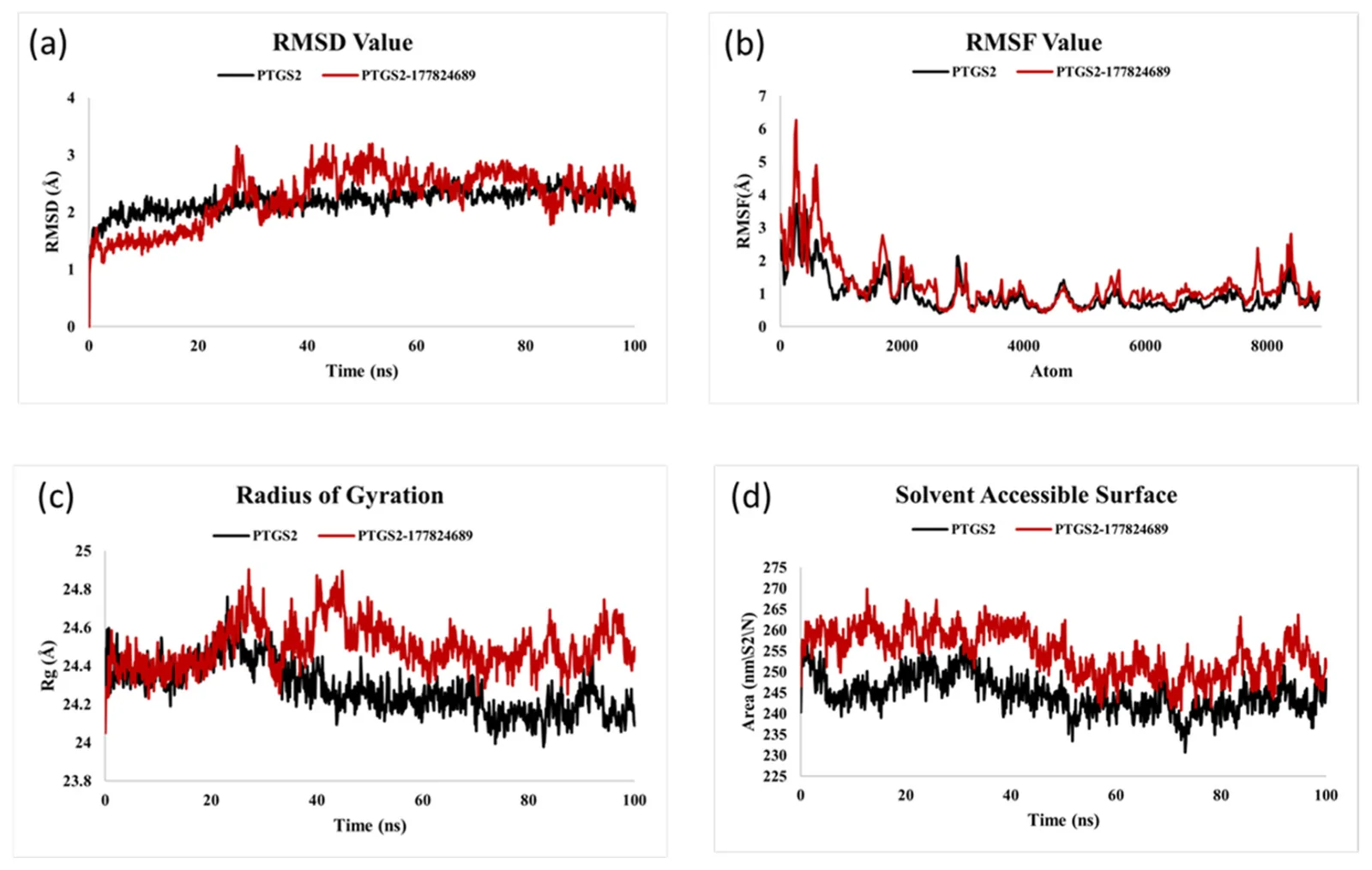

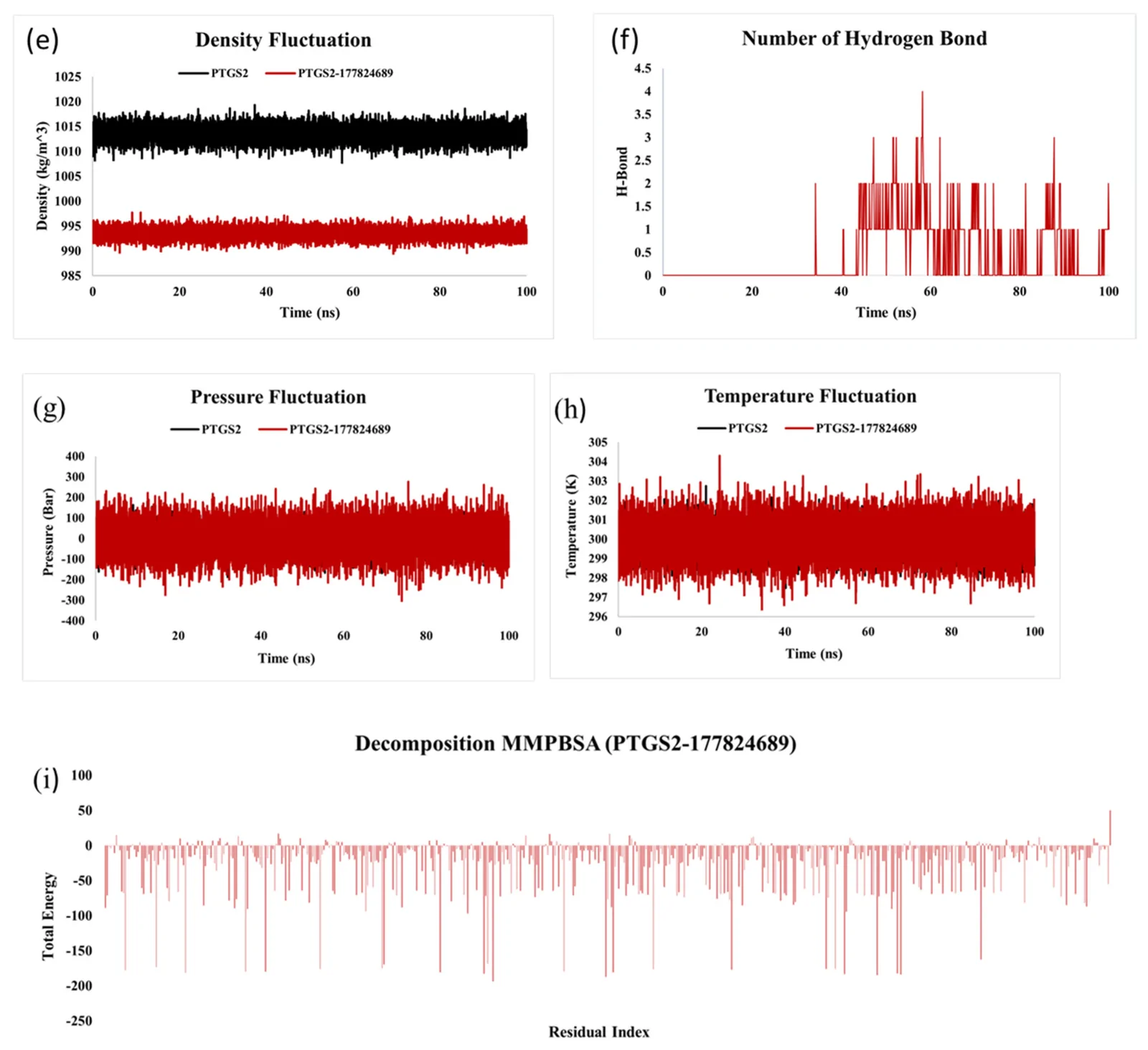
(**a**) RMSD; (**b**) RMSF; (**c**) radius of gyration; (**d**) SASA; (**e**) density of apo PTGS2 and PTGS2–Caffeyol pinoresinol complex (Kg/m^3^); (**f**) hydrogen bonding of PTGS2–Caffeyol pinoresinol complex; (**g**) pressure fluctuaiton; (**h**) temperetaure fluctuation; (**i**) decomposition mmpbsa (Kcal/mol).

Molecular dynamics simulations of the PTGS2–Caffeyol pinoresinol complex over 100 ns (Figure 9) were performed to evaluate structural behavior in comparison with the apo protein [45,46]. Both systems reached equilibrium at around 20 ns, with stable trajectories thereafter. The ligand-bound complex shows slightly higher RMSD values, indicating mild conformational adjustments upon Caffeyol pinoresinol binding, while overall structural integrity is maintained. RMSF analysis reveals low flexibility for most residues, with higher fluctuations in loop regions and terminal residues. In the ligand-bound system, localized increases in flexibility near the binding site suggest that ligand interaction induces controlled structural adjustments without destabilizing the protein. Figure 9g shows that the system pressure remained centered around the target value of 1 bar with only normal instantaneous fluctuations throughout the 100 ns simulation, indicating stable pressure equilibration under NPT conditions. Figure 9h demonstrates that the system temperature was consistently maintained around 300 K with only minor fluctuations, confirming effective thermal equilibration. Figure 9i shows residue-wise MM/PBSA energy decomposition, where most active-site residues exhibited negative energy contributions, with the strongest favorable interactions approaching approximately −200 kJ/mol, highlighting their significant role in ligand stabilization.

**Figure 10:**
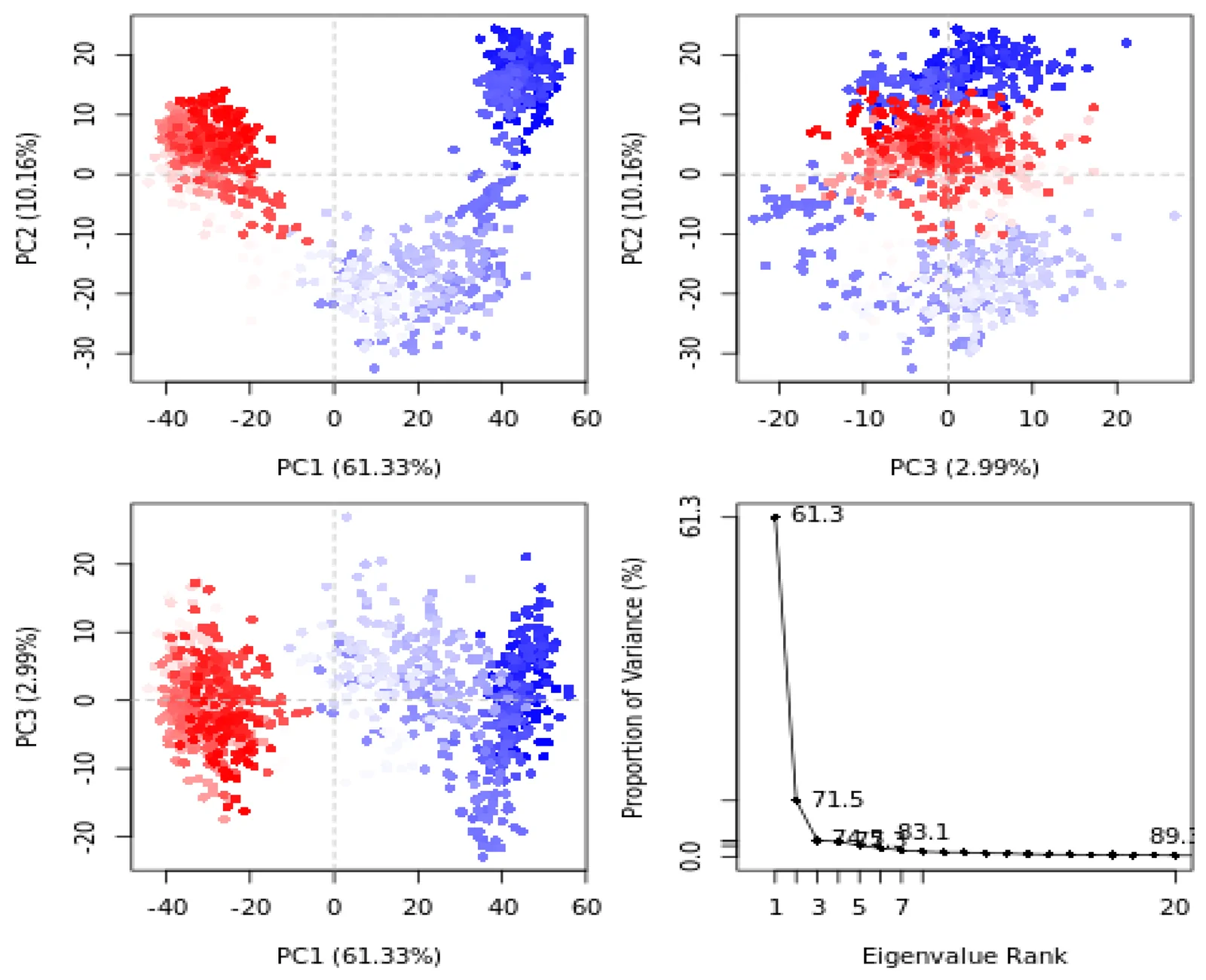
Principle component analysis of PTGS2–Caffeyol pinoresinol.

The radius of gyration (Rg) analysis indicates that apo PTGS2 maintains a stable, compact structure, while the PTGS2–Caffeyol pinoresinol complex shows a slight, controlled expansion upon ligand binding, reflecting minor conformational adjustments without compromising overall stability. Solvent accessible surface area (SASA) values are higher in the complex, suggesting increased residue exposure due to subtle structural rearrangements, yet remain within a stable range. System density profiles confirm proper equilibration, with only minor differences between apo and complex systems. Hydrogen bond analysis reveals intermittent but recurring interactions, contributing to ligand stabilization within the binding pocket. Principal component analysis (PCA) (Figure 10) demonstrates that the complex samples a defined conformational space with controlled flexibility, indicating dynamic stability without major structural transitions.

Overall, the simulation highlights that Caffeyol pinoresinol forms a stable and adaptable complex with PTGS2, maintaining consistent structural parameters and sustained intermolecular interactions, supporting its potential as a promising inhibitor candidate.

### 2.6. Principal Component Analysis (PC1)

Principal component analysis (PCA) of the molecular dynamics trajectory showing the conformational sampling of the system. The trajectory is color-coded according to simulation time, with red representing the initial frames and blue representing the final frames. The scree plot shows that the first principal component (PC1) contributes the majority of the motion (~61.3%), while subsequent components contribute significantly less. This sharp decline in eigenvalues indicates that the essential dynamics of the system are dominated by a single major motion, with the remaining motions representing minor fluctuations. Collectively, the PCA results suggest that the PTGS2–Caffeyol pinoresinol complex exhibits stable and well-defined dynamic behavior, with limited conformational variability and no evidence of large-scale structural instability.

Importantly, these simulations should be interpreted as computational stability assessments of a docking-prioritized ligand–target pair rather than confirmation that Caffeoyl pinoresinol is the experimentally validated mediator of the antioxidant activity observed for *A. atroviolaceum* extracts. Because the strongest antioxidant responses were observed in flower and leaf extracts rather than the bulb extract associated with Caffeoyl pinoresinol, the MD findings are more appropriately viewed as support for the plausibility of this predicted PTGS2 interaction than as evidence linking the compound directly to the measured antioxidant behavior of the most active organs.

### 2.7. DFT Analysis

Following docking and molecular dynamics analyses, Caffeoyl pinoresinol was subjected to density functional theory (DFT) and ADMET evaluation as a representative computationally prioritized ligand. These analyses were performed to characterize the electronic features and predicted pharmacokinetic/toxicity profile of the selected compound and thereby provide an additional context for its potential molecular behavior. As with the docking and simulation studies, these results should be interpreted as descriptors of a selected in silico candidate rather than as direct evidence of biological efficacy in the antioxidant assays.

**Figure 11:**
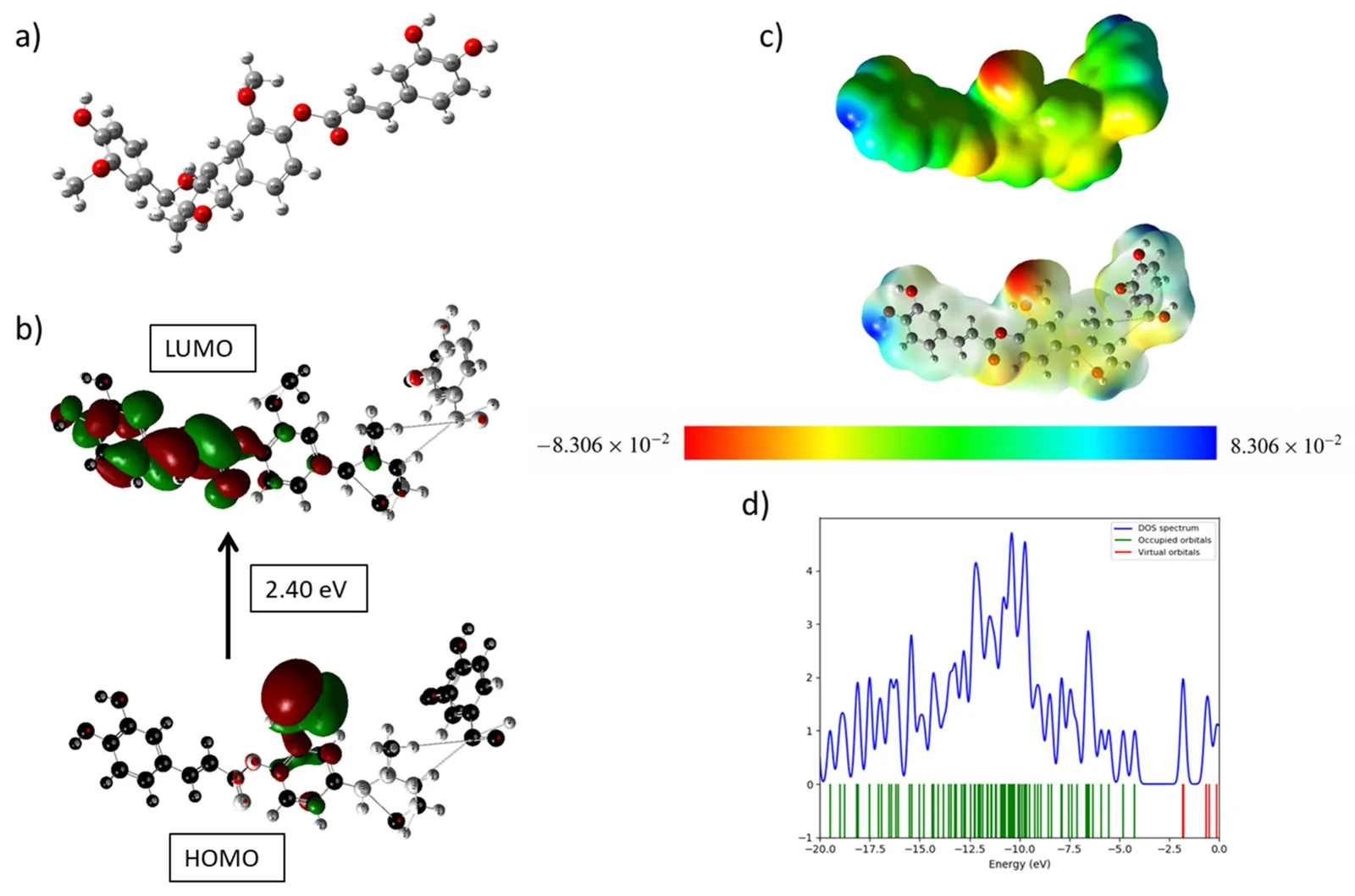
Density functional theory (DFT) analysis of Caffeoyl pinoresinol showing (**a**) optimized molecular structure, (**b**) frontier molecular orbitals and HOMO–LUMO energy gap, (**c**) molecular electrostatic potential (MEP) surface, and (**d**) density of states (DOS) spectrum.

DFT analysis of Caffeoyl pinoresinol (Figure 11) was conducted to examine its optimized geometry and electronic properties, which are essential for predicting molecular stability and reactivity [47]. The optimized 3D structure, generated using Gabedit, shows a stable aromatic framework with hydroxyl and ether groups, capable of hydrogen bonding and π–π interactions. This lowest-energy conformation reflects structural stability and provides a basis for further electronic property analysis, including frontier molecular orbitals, molecular electrostatic potential (MEP), and density of states (DOS) (Figure 11). Conversely, the lowest unoccupied molecular orbital (LUMO) is distributed over adjacent conjugated regions, suggesting potential sites for electron acceptance. The calculated HOMO–LUMO energy gap of 2.40 eV indicates moderate chemical reactivity and good molecular stability. Such a relatively small band gap facilitates charge transfer interactions and suggests that the compound can participate effectively in intermolecular interactions within biological environments.

The molecular electrostatic potential (MEP) map shown in Figure 11c highlights the charge distribution across the molecular surface. Regions colored in red represent areas of negative electrostatic potential, typically associated with oxygen atoms and electron-rich functional groups, which may serve as potential sites for electrophilic attack or hydrogen bond acceptors. In contrast, the blue regions indicate positive electrostatic potential, generally corresponding to hydrogen atoms attached to electronegative atoms and acting as potential hydrogen bond donors. The distribution of these electrostatic regions suggests favorable interaction sites that could contribute to ligand binding with biological targets.

Finally, the density of states (DOS) plot in Figure 11d illustrates the distribution of electronic energy levels within the molecule. The occupied orbitals appear predominantly in the negative energy region, while the virtual orbitals are located closer to the Fermi level. The separation between these energy levels further supports the calculated HOMO–LUMO gap and reflects the electronic stability of the compound. The DOS profile also indicates the presence of multiple closely spaced energy states, which may facilitate electron transitions and contribute to the molecule’s chemical reactivity. Overall, the DFT analysis demonstrates that Caffeoyl pinoresinol possesses a stable optimized structure, moderate electronic reactivity, and favorable charge distribution, supporting its potential to engage in strong intermolecular interactions with biological targets.

However, these electronic descriptors should not be interpreted as direct proof of antioxidant or PTGS2-modulating activity in the biological sense. Rather, they provide a theoretical explanation for why Caffeoyl pinoresinol may be capable of engaging in stabilizing interactions within a protein-binding environment, thereby supporting its selection for further exploratory study.

### 2.8. ADMET Analysis

Caffeoyl pinoresinol was subjected to a post-docking evaluation to assess its pharmacokinetic properties and safety profile (Table 3) in relation to its predicted binding potential. The compound has a molecular mass of 520.53 g/mol and a moderate iLogP of 3.90, indicating balanced lipophilicity that favors membrane permeability while avoiding excessive hydrophobicity. Its 3 hydrogen bond donors, 9 acceptors, and a TPSA of 123.91 Å^2^ suggest a strong capacity for stable hydrogen bonding, while 8 rotatable bonds confer sufficient flexibility to adapt within diverse protein binding pockets. Although its water solubility is low (2.87 × 10^−3^ mg/mL, Log S = −6.06), the molar refractivity of 138.04 supports effective steric complementarity and polarizability, facilitating robust interactions with target proteins. Toxicity profiling places the compound in class 4 with an LD50 of 1500 mg/kg, reflecting low acute toxicity, and shows inactivity toward mutagenicity, cytotoxicity, hepatotoxicity, thyroid receptor beta, estrogen receptor alpha, neurotoxicity, and cardiotoxicity, although potential carcinogenic activity indicates that long-term exposure should be carefully evaluated.

Taken together, the DFT and ADMET results provide additional supportive computational characterization of Caffeoyl pinoresinol after its prioritization in the docking screen. Nevertheless, these analyses do not resolve the central biological limitation of the present study: Caffeoyl pinoresinol was selected because of its predicted PTGS2-binding behavior, whereas the experimental antioxidant assays did not directly identify it as the principal determinant of the strongest extract activities. Accordingly, the DFT and ADMET findings should be viewed as useful descriptors of a candidate ligand emerging from the in silico workflow, not as evidence that the compound is the validated active antioxidant principle of *A. atroviolaceum*.

## 3. Materials and Methods

### 3.1. Plant Materials and Sample Preparation

**Figure 12:**
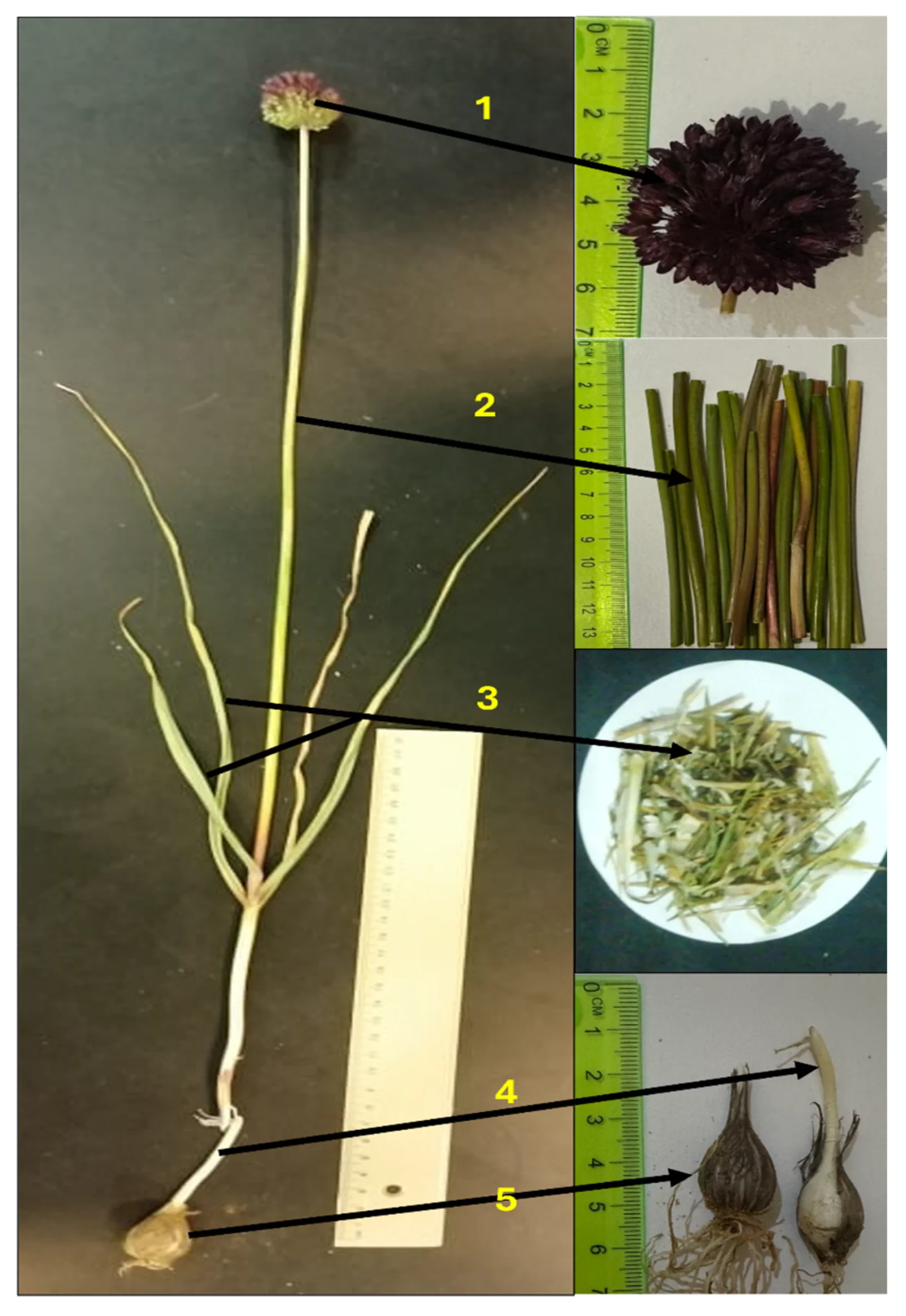
Whole plant specimens of *A. atroviolaceum* Boiss. collected from Al Nafud Alkabir (Saudi Arabia Kingdom). Legend: inflorescence with dark-purple–violet flower (1); flower stalk (2); linear, flattened, non-fistulated leaves (3); pseudostem (4); ovoid–globular bulb with gray–brown tunic (5). (Photos taken by Prof. Mejdi Snoussi).

*A. atroviolaceum* plant specimens were harvested in July 2022 from Al Nafud Alkabir (27°53′38.3″ N 40°27′14.4″ E) located at the north-western region of Saudi Arabia. The collected plant material (Figure 12) was botanically identified, and Voucher specimen (AN 009) was deposited in the Faculty of Science in the Biology Department. Fresh organs were washed with sterile distilled water to remove sand and debris and cut into small pieces. Each extraction was performed using 20 g of fresh plant material and 200 mL of ethanol (solid-to-solvent ratio: 1:10, *w*/*v*) at room temperature for 48 h. The extraction was repeated three times under identical conditions. The combined extracts were filtered and concentrated under reduced pressure using a rotary evaporator at 40 °C, and the resulting dried extracts were transferred to sterile amber vials and stored at 4 °C until further analysis. The yield of extraction (%, *w*/*w*) was calculated and was about 19.52 ± 1.29% for bulbs, 6.52 ± 0.58% for flowers, 8.52 ± 0.79% for leaves, and 10.36 ± 0.98% for the flower stalk.

### 3.2. Total Phenolic Content (TPC), Total Flavonoid Content (TFC), and Total Tannin Content (TTC)

EAAE was investigated for its phytochemical composition across different plant organs, and the total phenolic content of its ethanolic extracts was determined using the Folin–Ciocalteu assay as previously described [48]. Total flavonoid content was quantified according to the procedure outlined in [49], while total tannin content was estimated following the method reported by Haddaji et al. [50].

### 3.3. UHPLC-QTOF-IMS Analysis

Chromatographic analysis was performed on a ACQUITY UHPLC system coupled with a Vion IMS QToF mass spectrometer equipped with an electrospray ionization source (ESI) (Waters Corp., Milford, MA, USA). Separation was achieved using an ACQUITY UHPLC HSS T3 column (100 mm × 2.1 mm; 1.8 µm particle size) maintained at 30 °C, with a flow rate of 0.3 mL/min and an injection volume of 5 µL. The mobile phases were 0.1% formic acid in water (A) and 0.1% formic acid in acetonitrile (B); running a gradient in the ranges of 2–25% B (0–1 min), 25–50% B (1–5 min), 50–75% B (5–7 min), and 75–90% B (7–9 min); holding at 95% B (9–13 min); and resetting to 2% B (13–15 min). Mass spectrometry was operated in the *m*/*z* range of 50–1200 (scan time of 0.150 s) with capillary and cone voltages set at 2.2 KV and 50 V, and source and desolvation temperatures of 130° and 350 °C, respectively. Nitrogen (N_2_) gas flows were maintained at 50 L/h (cone) and 800 L/h (desolvation), while mass accuracy was calibrated using leucine/enkephalin (100 pg/µL). Data processing and characterization were completed via online UNIFI software (version 3.0.0) using the Traditional Chinese Medicine System Pharmacology (TCMSP), PubChem, MassBank, and ChemSpider databases. Annotation was based on the combined matching of accurate precursor mass, characteristic MS/MS fragmentation patterns, isotopic distribution, and ion-mobility-derived collision cross-section (CCS) values against these spectral libraries. As confirmation with authentic standards was not performed, all reported metabolites (excepting Bisdemethoxycurcumin, MSI level 3, and quercetin MSI level 1) correspond to level 2 (putatively annotated compounds) according to the Metabolomics standards Initiative (MSI) reporting criteria [51,52,53].

### 3.4. Antioxidant Capacities

The DPPH radical scavenging activity was evaluated according to the protocol, with slight modifications, by mixing a methanolic DPPH• solution with different concentrations of the essential oil, followed by incubation in the dark for 30 min and measurement of absorbance at 515 nm. The percentage of radical scavenging activity (SA%) was calculated, and IC_50_ values were determined as the concentration required to inhibit 50% of DPPH radicals. Analyses were carried out in three replicates.

The ABTS^+^• radical scavenging capacity of the extract was assessed following the method of Braca et al. [54] with slight modifications, over a concentration range of 0–2 mg/mL. The ABTS^+^• solution was generated by reacting ABTS with potassium persulfate in the dark for 12 h. The extract was then mixed with the ABTS^+^• solution, incubated for 6 min, and the absorbance measured at 734 nm. Butylated hydroxytoluene (BHT) was used as a standard, and the percentage of radical scavenging activity was determined. All experiments were conducted in triplicate.

ABTS+ radical scavenging activity (%) = [(Acontrol − Asample)/Acontrol] × 100; where Acontrol is the absorbance of the control reaction and Asample is the absorbance of the extract.

The inhibition of *β*-carotene oxidation by peroxyl radicals in the different organ extracts was evaluated using the protocol described [55]. Briefly, a β-carotene solution was combined with linoleic acid, Tween 20, and plant organ extracts, and chloroform was removed by rotary evaporation at 30 °C for 20 min. Distilled water was then added, and the mixture was vigorously shaken to form a stable emulsion. Aliquots of the emulsion were incubated in a water bath at 50 °C for 2 h, and absorbance was measured at 470 nm at 0 and 2 h. Antioxidant activity was expressed as the percentage inhibition of *β*-carotene degradation relative to the control, and all measurements were performed in triplicate.

The ferric-reducing antioxidant power (FRAP) assay was used in order to assess the ferric (Fe^3+^)-reducing ability of the tested extracts following the method described by Yildirim et al. [56]. The extract was mixed with phosphate buffer (0.2 M, pH 6.6) and potassium ferricyanide 1%, incubated at 50 °C for 30 min, and the reaction was stopped with trichloroacetic acid 10%. After centrifugation at 3000 rpm for 10 min, the supernatant was combined with ferric chloride (FeCl_3_)-1%, incubated for 10 min at room temperature, and absorbance was then measured at 700 nm. An increase in absorbance indicated greater reducing power, with Butylated hydroxytoluene (BHT) used as a reference standard. All analyses were performed in triplicate.

### 3.5. Network Pharmacology Analysis

Target prediction and network pharmacology analysis: A total of 63 phytochemicals from *A. atroviolaceum* were screened for potential therapeutic targets. Compound structures were obtained from PubChem, and target prediction was performed using SwissTargetPrediction and PharmMapper, with validation via UniProt using smiles id [57,58]. Disease-related targets associated with obesity and gastrointestinal disorders were retrieved from GeneCards, OMIM, and DisGeNET [59,60,61]. Overlapping targets were identified using FunRich and Venny, and key targets including TNF, JUN, HRAS, ALB, MAPK1, PTGS2, TP53, BCL2, EGFR, and TGFB1 were selected, with 13 compounds chosen for docking studies [62].

PPI network and enrichment analysis: Protein–protein interaction (PPI) data were obtained from STRING (confidence score ≥ 0.400) and visualized using Cytoscape software (version 3.8.2), where topological parameters (degree, betweenness, and closeness centrality) were used to identify hub proteins [63]. Hub gene identification was performed using the cytoHubba plugin, while functional enrichment analysis was conducted using DAVID and ShinyGO [64,65]. Significant Gene Ontology (GO) terms and KEGG pathways (FDR < 0.05) were visualized using SRplot [66].

Protein and ligand preparation: Protein structures were obtained from the RCSB Protein Data Bank and prepared by removing heteroatoms and water molecules using Biovia Discovery Studio, followed by energy minimization with Swiss-PdbViewer [66,67]. Ligands were processed using Open Babel and PyRx, and energy-minimized using the Ghemical force field [68,69].

### 3.6. In Silico Analysis

Molecular docking: The top 13 compounds were docked with nine selected targets, TNF (pdb id: 4ZCH), JUN (pdb id: 1JNM), HRAS (pdb id: 121P), ALB (pdb id: 1N5U), MAPK1 (pdb id: 2Y9Q), PTGS2 (pdb id: 5F19), TP53 (pdb id: 2G3R), BCL2 (pdb id: 2W3L), EGFR (pdb id: 3P0Y), and TGFB1 (pdb id: 5VQP), using AutoDock Vina software (version 4.2) in PyRx to investigate interactions relevant to inflammatory diseases (“Docking-Based Virtual Screening Using PyRx Tool,” 2021) [70]. The three-dimensional structure of the target protein was prepared by removing co-crystallized ligands and water molecules, followed by the addition of polar hydrogen atoms and Kollman charges. The prepared protein was saved in PDBQT format for docking. Ligand structures were obtained from PubChem, energy-minimized, protonated, and converted to PDBQT format using Open Babel integrated within PyRx. The docking search space was defined using a grid box centered at X = 20.8612, Y = 37.5501, and Z = 59.3402 Å, with dimensions of 64.86 × 73.30 × 57.94 Å (X × Y × Z), ensuring complete coverage of the active binding site. The exhaustiveness parameter was set to 8, while all other parameters were maintained at their default AutoDock Vina settings. Binding affinities were estimated using the AutoDock Vina scoring function, and the best-ranked binding pose with the lowest predicted binding free energy (kcal/mol) was selected for further interaction analysis. To validate the docking protocol, the co-crystallized ligand was re-docked into the protein binding pocket using the same docking parameters. The reproduced binding pose showed a root-mean-square deviation (RMSD) of less than 2.0 Å compared with the crystallographic conformation, confirming the reliability and accuracy of the docking protocol. Docking was focused on protein active sites to maximize binding affinity. Binding scores were used to evaluate docking outcomes, and PyMol visualized binding conformations. Hydrogen-bonding and non-bonded interactions were further analyzed with Discovery Studio [71].

Molecular dynamics simulation: Molecular dynamics simulations were carried out using GROMACS 2022 in a Linux Ubuntu environment to evaluate the stability of the apo protein and protein–ligand complexes over a 100 ns trajectory [72]. The CHARMM36 force field and TIP water model were applied under physiological conditions (300 K, 1 bar) [73]. Following energy minimization and NVT/NPT equilibration with positional restraints, production simulations were performed. Structural stability was assessed using RMSD, RMSF, radius of gyration (Rg), solvent-accessible surface area (SASA), and hydrogen bond analysis.

DFT calculation: Calculations were performed using Gaussian software (version 16) at the B3LYP/6-31G level to investigate electronic properties. Frontier molecular orbital analysis was used to determine HOMO–LUMO energy gaps [74], while electrostatic potential analysis provided insights into charge distribution. Visualization and analysis were conducted using Multiwfn 3.6, GaussSum, VMD, and gnuplot [75,76].

Overall, the integrated experimental and computational analyses provide a comprehensive characterization of the phytochemical composition and antioxidant potential of different organs of *Allium atroviolaceum*. The in vitro antioxidant assays demonstrated organ-specific antioxidant activities, while network pharmacology, molecular docking, molecular dynamics, DFT, and ADMET analyses were employed to computationally prioritize potential bioactive phytochemicals and their putative molecular targets. Although several prioritized compounds are glycosylated phytochemicals that may exhibit limited oral bioavailability because of their relatively high molecular weight and polarity, they represent the experimentally identified constituents of the extracts and therefore constituted the basis of the computational analyses. Their corresponding aglycones, which may arise through metabolic deglycosylation in vivo, were beyond the scope of the present study and warrant future investigation. Accordingly, the computational findings should be regarded as hypothesis-generating and provide a rational foundation for subsequent biochemical, pharmacokinetic, and cell-based validation.

### 3.7. Data Analysis

All assays were conducted in triplicate. Experimental data were processed using GraphPad Prism v10.4 (GraphPad Software Inc., Boston, MA, USA). Group comparisons were performed using two-way ANOVA followed by Tukey’s post hoc test, with significance defined as *p* < 0.05.

## 4. Conclusions

In conclusion, this work uncovers organ-specific variations in the phytochemical composition of *A. atroviolaceum* and shows that it is a rich source of phenolic compounds with notable in vitro antioxidant activity. UHPLC-QTOF-IMS profiling revealed organ-specific phytochemicals, including flavonoids, anthocyanins, and phenolics, while in vitro assays confirmed significant antioxidant activity. A combined in silico approach, encompassing network pharmacology, molecular docking, ADMET prediction, molecular dynamics, and DFT analysis, identified several putative targets (EGFR, HRAS, TGFB1, BCL2, JUN, ALB, TNF, TP53, MAPK1, and PTGS2) potentially associated with oxidative stress, inflammation, and apoptosis. Potential bioactive substances and molecular targets linked to oxidative stress were found by the integrated computational analysis; nevertheless, these results are hypothesis-generating and need to be verified experimentally. Overall, this study lays the groundwork for further research to determine which plant organs have the highest concentrations of antioxidant components and to validate the substances and processes responsible for the biological activity that has been seen.

## Figures and Tables

**Table 1:** Total phenolic, flavonoid, and tannin contents of bulbs, flower stalks, leaves, and flowers. Results are reported as the mean ± SD of three experiments (n = 3). Different letters indicate mean values significantly different at *p* < 0.05, according to a one-way ANOVA followed by Tukey’s post hoc test.

	Bulbs (BE1)	Flower Stalks (BE2)	Leaves (BE3)	Flowers (BE4)
Total Phenolic Content (mg GAE/g extract)	59.40 ± 0.192	72.86 ± 1.864	58.04 ± 2.635	103.50 ± 0.192
Total Flavonoid Content (mg QE/g extract)	37.25 ± 4.36	53.25 ± 3.417	60.16 ± 6.128	108.00 ± 5.656
Total Tannin Content (mg TAE/g extract)	39 ± 0.707	27.5 ± 2.828	30.5 ± 1.414	82.85 ± 1.909

**Table 2:** Tentative identification of the metabolites of AAE (bulbs, flower stalks, leaves, and flowers) using the UHPLC-QTOF-IMS technique.

N°	RT (min)	Calc. *m*/*z*	Exp. *m*/*z*	Error (ppm)	Tentative Identification	Formula	Bulbs (%)	Flower Stalks (%)	Leaves (%)	Flowers (%)	Identification Level (MSI Level)
1	0.53	441.0865	441.0870	1.13	Catechin-7-O-gallate	C_22_H_18_O_10_		5.067			2
2	0.56	377.3197	377.3169	−7.42	Caffeoyl-6-O-hexoside dihydrate 1	C_15_H_22_O_11_	31.519	22.551			2
3	0.61	549.1672	549.1679	0.91	Liquiritin apioside	C_26_H_30_O_13_	11.506	3.791	0.869	1.174	2
4	0.89	285.0447	285.0452	1.75	Luteolin	C_15_H_10_O_6_	5.852		-		2
5	0.97	267.0693	267.0695	0.75	Formononetin	C_16_H_12_O_4_				0.318	2
6	1.55	329.0605	329.0613	1.52	Quercetin-3,4′-dimethyl ether	C_17_H_14_O_7_	0.833				2
7	1.93	579.1727	579.1734	0.89	Naringin	C_27_H_32_O_14_	18.455				2
8	2.07	787.1996	787.1988	0.92	Moracetin	C_33_H_40_O_22_			4.864		2
9	2.08	515.1188	515.1169	−3.6	3,4-dicaffeoylquinic acid	C_25_H_24_O_12_	13.125	1.100		0.517	2
10	2.09	323.1301	323.1306	1.55	Glabridin	C_20_H_20_O_4_		-		0.384	2
11	2.14	771.2048	771.2043	1.3	Kaempferol 3-O-*β*-D-glucosyl(1->2)-*β*-D-galactoside 7-O-*β*-D-glucoside	C_33_H_40_O_21_	0.051		1.284		2
12	2.16	625.1446	625.1451	0.8	Quercetin 3-sophoroside	C_27_H_30_O_17_		0.722	9.339		2
13	2.20	609.1472	609.1466	0.89	Multinoside A	C_27_H_30_O_16_			3.337		2
14	2.22	610.1506	610.1512	0.98	Cyanidin 3-gentiobioside	C_27_H_31_O_16_^+^	0.101		3.238	4.300	2
15	2.28	377.3197	377.3169	−7.42	Caffeoyl- 6-O-hexoside dihydrate 2	C_15_H_22_O_11_	0.136				2
16	2.30	355.1058	355.1037	2.53	Ferulic acid *β*-glucoside 1	C_16_H_20_O_9_	1.176				2
17	2.37	625.1403	625.1409	0.96	Quercetin-7-O-*β*-D-glucopyranosyl(1->6)-*β*-D-glucopyranoside	C_27_H_30_O_17_			0.692	0.307	2
18	2.43	625.1423	625.1428	0.9	Quercetin 3,5-digalactoside	C_27_H_30_O_17_		0.881	7.227	4.301	2
19	2.44	609.1454	609.1424	−4.94	Quercetin-3-O-neohesperidoside	C_27_H_30_O_16_			3.637		2
20	2.50	639.1668	639.1639	4.54	Quercetin-3-O-*β*-D-galactopyranosyl-7-O-*β*-D-glucopyranoside	C_28_H_32_O_17_			0.001	0.523	2
21	2.55	609.1481	609.1486	0.82	Kaempferol-7-O-*β*-D-glucopyranosyl (1–>4)-*β*-D-glucopyranoside	C_27_H_30_O_16_		1.108			2
22	2.56	594.1555	594.1559	0.67	Cyanidin 3-rutinoside	C_27_H_31_O_15_^+^				1.635	2
23	2.59	461.0704	461.0698	1.3	Kaempferol 3 glucuronide	C_21_H_18_O_12_			0.433		2
24	2.6	463.0831	463.0836	1.08	Hyperoside	C_21_H_20_O_12_		4.516	36.597		2
25	2.62	623.1632	623.1637	0.8	Quercetin-3-O-α-L-rhamnopyranosyl-7-O-β-D-glucopyranoside	C_27_H_28_O_17_		1.342	1.155	0.482	2
26	2.63	593.1517	593.1520	0.51	Kaempferol-O-rhamnosyl hexoside	C_27_H_30_O_15_			0.971	1.635	2
27	2.72	448.0930	448.0925	1.12	Cyanidin 3-O-glucoside	C_21_H_21_O_11_^+^		4.291	14.439	6.624	2
28	2.73	447.0905	447.0914	2.01	Kaempferol 3-O-*β*-D-glucoside	C_21_H_20_O_11_				6.791	2
29	2.74	447.0882	447.0887	1.12	Luteolin 3′-glucoside	C_21_H_20_O_11_			Traces	Traces	2
30	2.81	431.0932	431.0937	1.02	Genistin	C_21_H_20_O_10_		1.454			2
31	2.83	431.064	431.0963	2.09	Kaempferol 7-O-rhamnoside	C_21_H_20_O_10_				1.262	2
32	2.86	462.1076	462.1081	1.08	Peonidin 3-glucoside	C_22_H_23_O_11_^+^		10.547	2.457	0.001	2
33	2.89	47.0878	475.0884	1.26	Luteolin-7-O-(6″-methylester)-*β*-D-glucuronide	C_22_H_20_O_12_				0.309	2
34	2.91	517.1913	517.1906	0.97	Caffeoyl pinoresinol	C_29_H_28_O_9_				4.878	2
35	2.94	505.0990	505.0995	0.99	Quercetin 3-(2″-acetylgalactoside)	C_23_H_22_O_13_				0.671	2
36	3.17	489.1015	489.1022	1.43	Kaempferol 3-O-(6”-O-acetyl)-*β*-D-glucopyranoside	C_23_H_22_O_12_				1.579	2
37	3.22	739.1936	739.1944	1.08	Procyanidin-B1-6-C-*β*-D-glucopyranoside	C_36_H_36_O_17_				0.581	2
38	3.46	285.0321	285.0326	1.12	Luteolin	C_15_H_10_O_6_	1.329	2.647	2.031	0.001	2
39	3.47	301.0302	301.0307	1.66	Quercetin	C_15_H_10_O_7_			2.872	1.113	1
40	3.95	269.0372	269.0378	2.23	Apigenin	C_15_H_10_O_5_		1.721	0.524	18.851	2
41	3.98	499.1225	499.1222	−0.60	Catechin pentaacetate	C_25_H_24_O_11_				0.278	2
42	4.02	329.0590	329.0595	0.81	3,3′-dimethylquercetin	C_17_H_14_O_7_		1.991		0776	2
43	4.03	577.1357	577.1366	1.56	Procyanidin B8	C_30_H_26_O_12_				1.083	2
44	4.05	300.0551	300.0557	2	Peonidin	C_16_H_13_O_6_^+^	3.537			5.399	2
45	4.06	286.0391	286.0398	2.45	Cyanidin	C_15_H_11_O_6_^+^				12.748	2
46	4.07	299.0481	299.0486	1.67	Hydroxygenkwanin	C_16_H_12_O_6_		9.301	1.206	5.402	2
47	4.16	315.0458	315.0453	1.59	Isorhamnetin	C_16_H_12_O_7_	0.763		2.921	14.188	2
48	4.38	623.1975	623.1981	0.96	Acteoside	C_29_H_36_O_15_			Traces	Traces	2
49	4.55	741.3186	741.3192	0.81	Cyanidin-3-O-xylosylrutinoside	C_32_H_389_O_20_	1.451				2
50	5.41	327.2170	327.2179	2.75	Trihydroxy octadecadienoic acid	C_18_H_32_O_5_	0.569				2
51	6.01	447.0926	447.0932	1.34	Kaempferol-7-O-*β*-D-glucopyranoside	C_21_H_20_O_11_	0.131				2
52	7.12	311.2293	311.2306	4.18	9-hydroperoxyoctadecadienoic acid	C_18_H_32_O_4_	8.555		Traces	Traces	2
53	8.74	433.2033	433.2075	9.23	Naringenin glucoside 1	C_21_H_22_O_10_	0.230		Traces		2
54	9.02	433.1154	433.1141	−3.00	Naringenin glucoside 2	C_21_H_22_O_10_			Traces	Traces	2
55	9.11	309.2901	309.2911	3.23	*p*-coumaric acid deoxyhexoside 3	C_15_H_18_O_7_	0.056				2
56	10.29	325.0923	325.0929	1.85	*p*-coumaric acid hexoside 1	C_15_H_18_O_8_			Traces	Traces	2
57	10.71	325.0923	325.0929	1.85	*p*-coumaric acid hexoside 2	C_15_H_18_O_8_	0.108				2
58	13.75	413.3834	413.3840	1.45	*β*-sitosterol	C_29_H_50_O		26.869			2
59	14.10	317.0296	317.0265	−9.78	Myricetin	C_15_H_10_O_8_			Traces	Traces	2
60	14.40	271.0602	271.0613	0.80	Naringenin	C_15_H_12_O_5_	0.453			Traces	2

**Table 3:** ADMET analysis of Caffeoyl pinoresinol.

Compound		Post-Docking Analysis	
	Name of Analysis	Properties	Values
Caffeoyl pinoresinol	ADME analysis	Mass (g/mol)	520.53
		iLogp	3.90
		H Donor	3
		H Acceptor	9
		Water Solubility (mg/mL)	2.87 × 10^−3^
		Rotational Bonds	8
		TPSA (Å^2^)	123.91
		Molar Refractivity	138.04
		Log S	−6.06
Caffeoyl pinoresinol	Toxicity profiling	Toxicity Class	4
		LD50 (mg/Kg)	1500
		Carcinogenicity	Active
		Mutagenicity	Inactive
		Cytotoxicity	Inactive
		Hepatotoxicity	Inactive
		Thyroid Hormone Receptor Beta (THRβ)	Inactive
		Estrogen Receptor Alpha (ER)	Inactive
		Neurotoxicity	Inactive
		Cardiotoxicity	Inactive

## Data Availability

The original contributions presented in this study are included in the article/Supplementary Materials. Further inquiries can be directed to the corresponding authors.

## Supplementary Materials

The following supporting information can be downloaded at: https://www.mdpi.com/article/10.3390/life16081256/s1. Supplementary Figure S1: UHPLC-QTOF-IMS base peak chromatograms of (A) bulbs, (B) stalks, (C) leaves, and (D) flowers. Supplementary Figure S2: MS and MS/MS fragmentation spectra of the principal metabolites identified by UHPLC-QTOF-IMS technique.
