# Supplementary material for: PTGS2-Based Network Pharmacology and Molecular Modeling Investigation of the Antioxidant Potential of Ethanolic Allium atroviolaceum Boiss. Extracts Across Plant Parts: Compositional Diversity, UHPLC-QTOF-IMS, and Experimental Validation
Source: Life (Basel). 2026 Jul 29;16(8):1256. doi: 10.3390/life16081256 (PMC13514844; doi:10.3390/life16081256)
Supplement: Supplementary file 1 [file life-16-01256-s001.zip › life-4375213-supplementary.pdf]

PTGS2-Based Network Pharmacology and Molecular Modeling Investigation of the Antioxidant Potential of *Ethanolic Allium atroviolaceum* Extracts across Plant Parts: Compositional Diversity, UHPLC-QTOF-IMS, and Experimental Validation

Mejdi Snoussi[1,2*], Emira Noumi[1,2], Manal Mohammed Alzahrani[3*], Khulood Fahad Alabbosh[1,2], Qusai Alsenani[1,2], Mamdouh Alshammari[1,2], Mohd Adnan[1,2], Arif Jamal Siddiqui[1,2], Riadh Ben Salah[4], Naourez Ktari[4,5], Karim Hosni[6], Vincenzo De Feo[7*], Adel Kadri[3,8*]

[1] Department of Biology, College of Science, University of Ha'il, P.O. Box 2440, Hail 81451, Saudi Arabia.
[2] Medical and Diagnostic Research Center, University of Ha'il, Ha'il 55473, Saudi Arabia.
[3] Department of Chemistry, Faculty of Science, Al-Baha University, Al-Baha 65527, Saudi Arabia.
[4] Laboratory of Microbial and enzymatic Biotechnologies and Biomolecules (LBMEB), Center of Biotechnology of Sfax (CBS), Road of Sidi Mansour Km 6, P.O. Box 1177, Sfax 3018, Tunisia.
[5] Higher Institute of Biotechnology of Sfax, Soukra Road km 4.5; BP. 261, 3038 Sfax – Tunisia.
[6] Laboratoire des Substances Naturelles, Institut National de Recherche et d'Analyse Physico-chimique (INRAP), Biotechpole de Sidi Thabet, 2090, Ariana, Tunisia.
[7] Department of Pharmacy. University of Salerno. Via Giovanni Paolo II. 132. Fisciano. 84084 Salerno, Italy.
[8] Faculty of Science of Sfax, Department of Chemistry, Sfax University, B.P. 1171, Sfax 3000, Tunisia.

* Corresponding Authors: Prof. Mejdi Snoussi, m.snoussi@uoh.edu.sa; Dr. Manal Mohammed Alzahrani mma.alzahrani@bu.edu.sa, Prof. Adel Kadri, lukadel@yahoo.fr

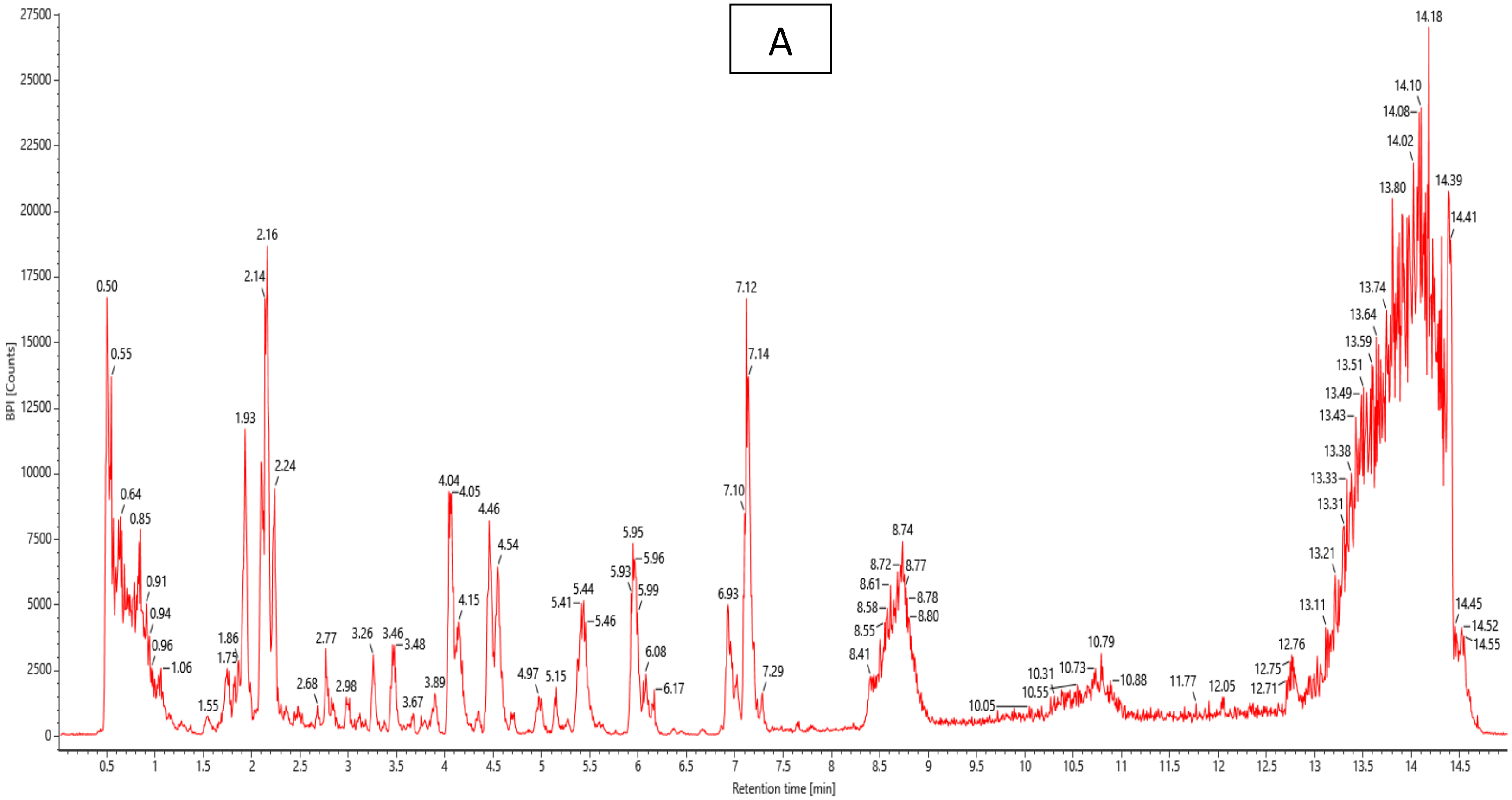
A
BPI [Counts]
Retention time [min]
27500
25000
22500
20000
17500
15000
12500
10000
7500
5000
2500
0
0.5
1
1.5
2
2.5
3
3.5
4
4.5
5
5.5
6
6.5
7
7.5
8
8.5
9
9.5
10
10.5
11
11.5
12
12.5
13
13.5
14
14.5
0.50
0.55
0.64
0.85
0.91
0.94
0.96
1.06
1.55
1.79
1.86
1.93
2.14
2.16
2.24
2.68
2.77
2.98
3.26
3.46
3.48
3.67
3.89
4.04
4.05
4.15
4.46
4.54
4.97
5.15
5.41
5.44
5.46
5.93
5.95
5.96
5.99
6.08
6.17
6.93
7.10
7.12
7.14
7.29
8.41
8.55
8.58
8.61
8.72
8.74
8.77
8.78
8.80
10.05
10.31
10.55
10.73
10.79
10.88
11.77
12.05
12.71
12.75
12.76
13.11
13.21
13.31
13.33
13.38
13.43
13.49
13.51
13.59
13.64
13.74
13.80
14.02
14.08
14.10
14.18
14.39
14.41
14.45
14.52
14.55

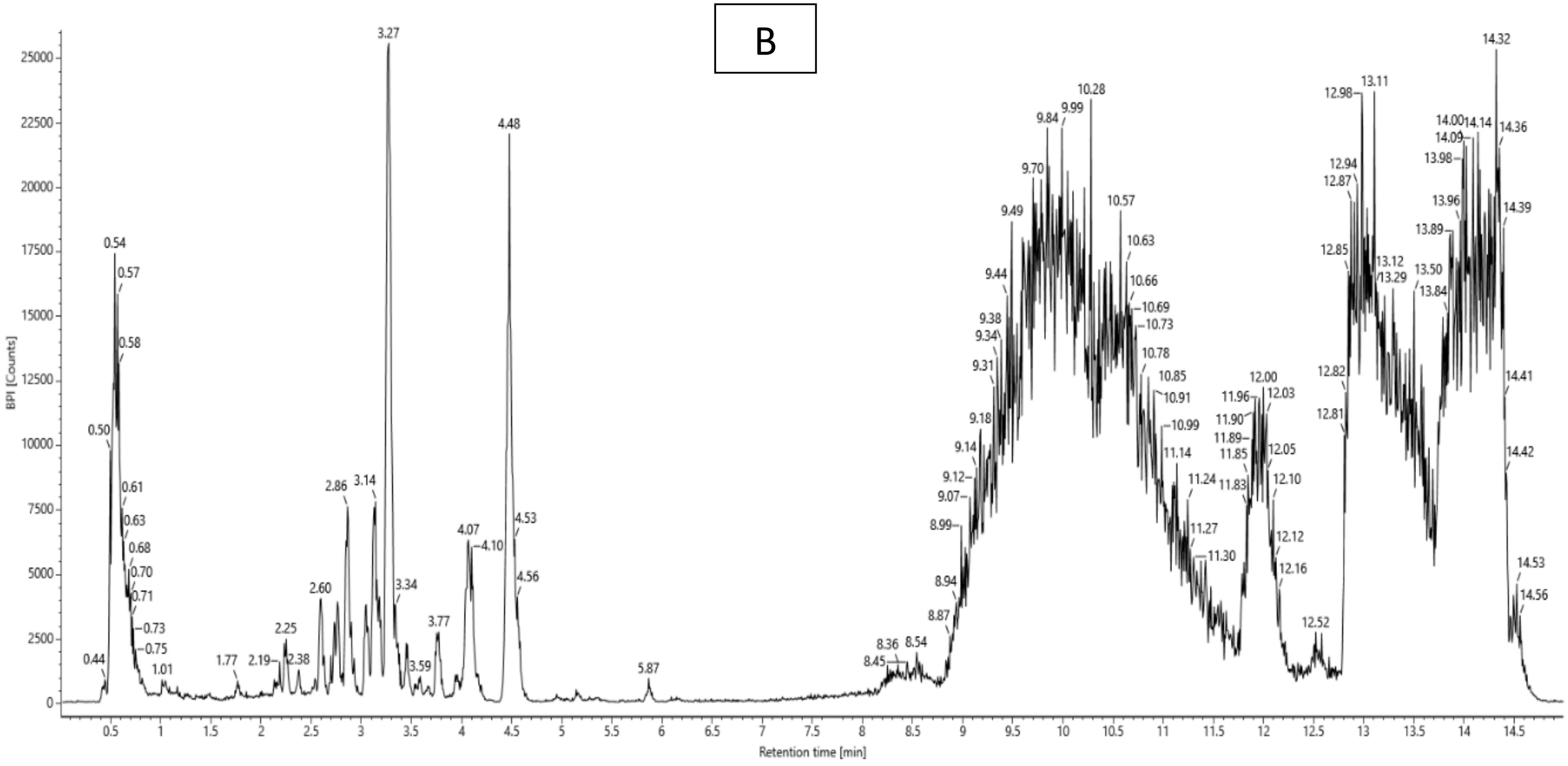
B
BPI [Counts]
25000
22500
20000
17500
15000
12500
10000
7500
5000
2500
0
Retention time [min]
0.5 1 1.5 2 2.5 3 3.5 4 4.5 5 5.5 6 6.5 7 7.5 8 8.5 9 9.5 10 10.5 11 11.5 12 12.5 13 13.5 14 14.5
0.44 0.50 0.54 0.57 0.58 0.61 0.63 0.68 0.70 0.71 0.73 0.75 1.01
1.77 2.19 2.25 2.38 2.60 2.86 3.14 3.27 3.34 3.59 3.77 4.07 4.10 4.48 4.53 4.56 5.87
8.36 8.45 8.54 8.87 8.94 8.99 9.07 9.12 9.14 9.18 9.31 9.34 9.38 9.44 9.49 9.70 9.84 9.99 10.28
10.57 10.63 10.66 10.69 10.73 10.78 10.85 10.91 10.99 11.14 11.24 11.27 11.30
11.83 11.85 11.89 11.90 11.96 12.00 12.03 12.05 12.10 12.12 12.16 12.52
12.81 12.82 12.85 12.87 12.94 12.98 13.11 13.12 13.29 13.50 13.84
13.89 13.96 13.98 14.00 14.09 14.14 14.32 14.36 14.39 14.41 14.42 14.53 14.56

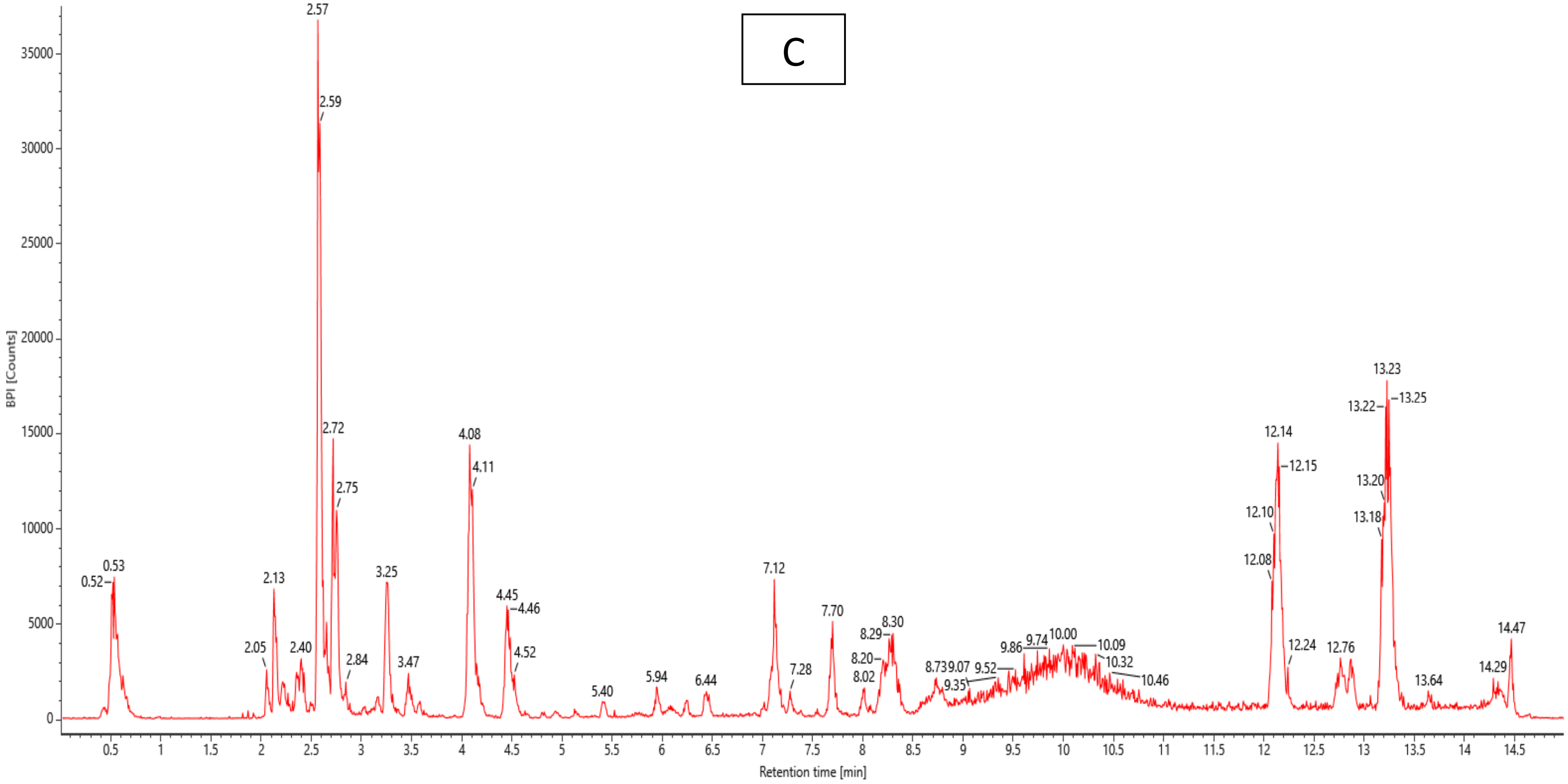
C
BPI [Counts]
Retention time [min]
2.57
2.59
2.72
2.75
2.84
0.52
0.53
2.05
2.13
2.40
3.25
3.47
4.08
4.11
4.45
4.46
4.52
5.40
5.94
6.44
7.12
7.28
7.70
8.02
8.20
8.29
8.30
8.73
9.07
9.35
9.52
9.86
9.74
10.00
10.09
10.32
10.46
12.08
12.10
12.14
12.15
12.24
12.76
13.18
13.20
13.22
13.23
13.25
13.64
14.29
14.47

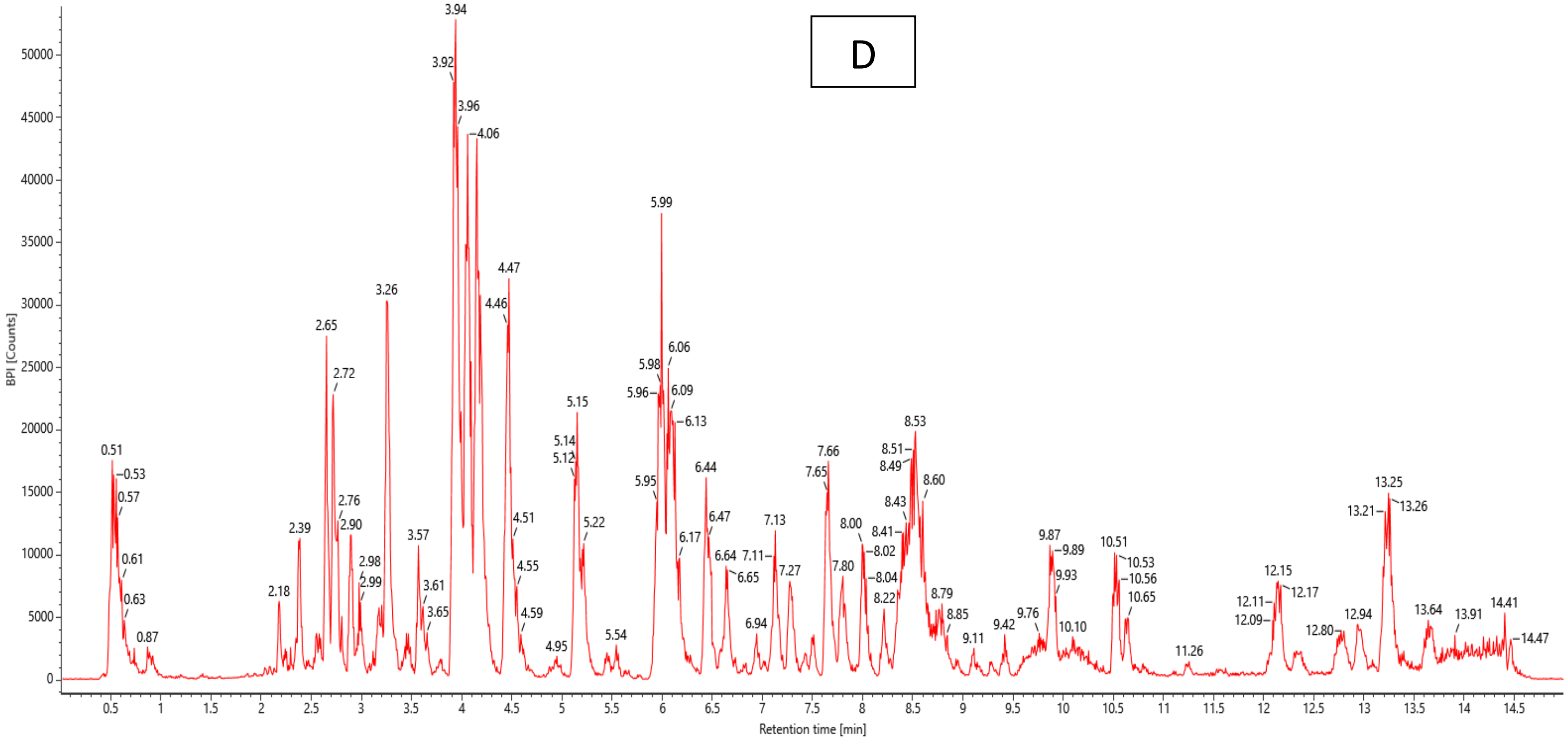

Supplementary FigureS1. UHPLC-QTOF-IMS base peak chromatograms of (A) bulbs, (B) stalks, (C) leaves, and (D) flowers.

Supplementary FigureS2. MS and MS/MS fragmentation spectra of the principal metabolites identified by UHPLC-QTOF-IMS technique.

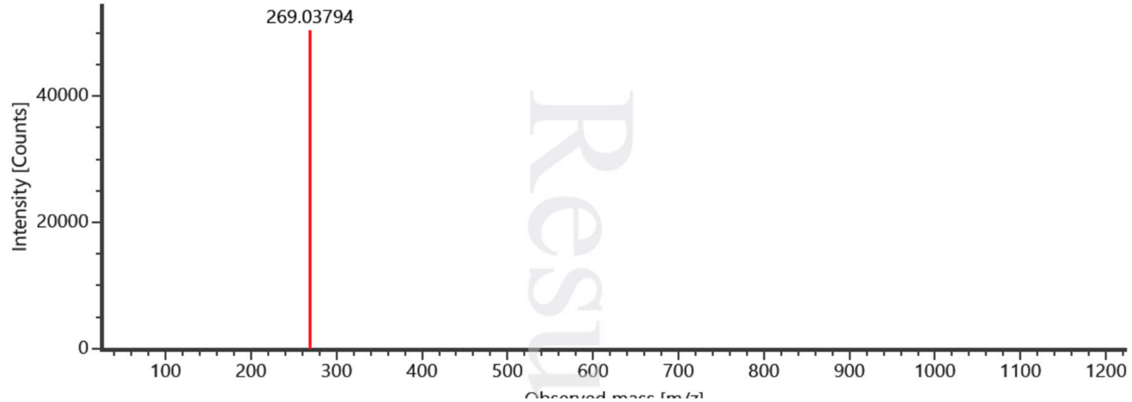

Apigenin

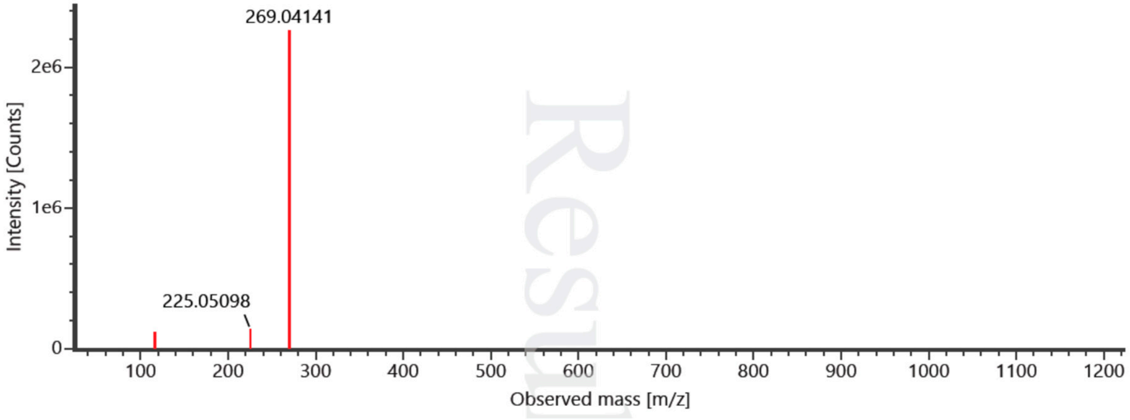

β-sitosterol

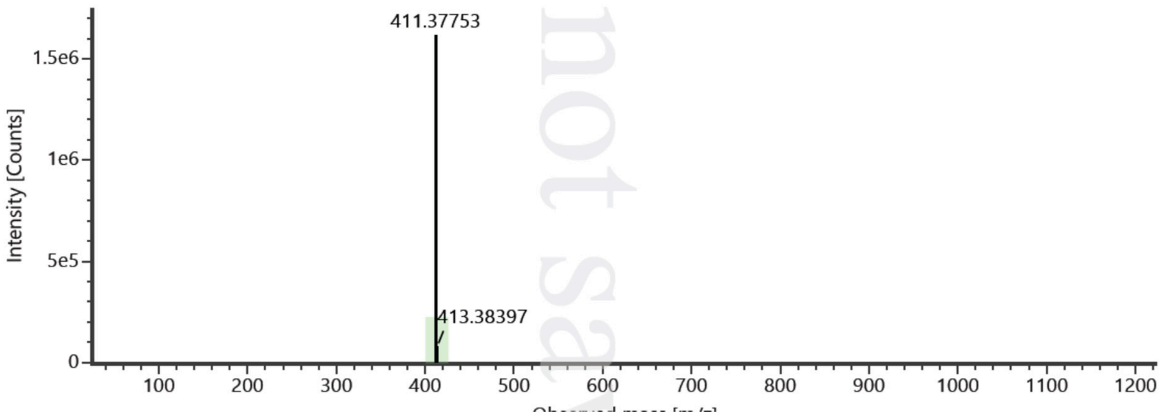

Cyanidin-3-O-glucoside

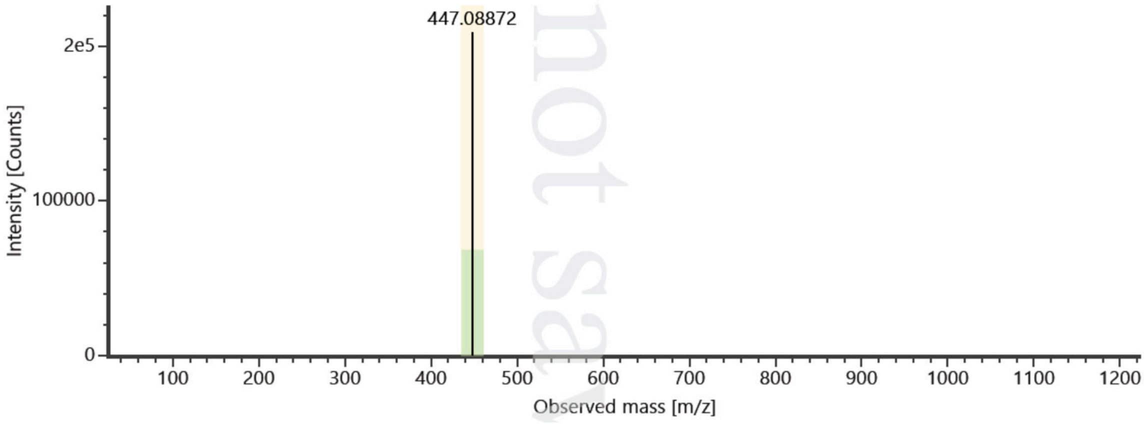

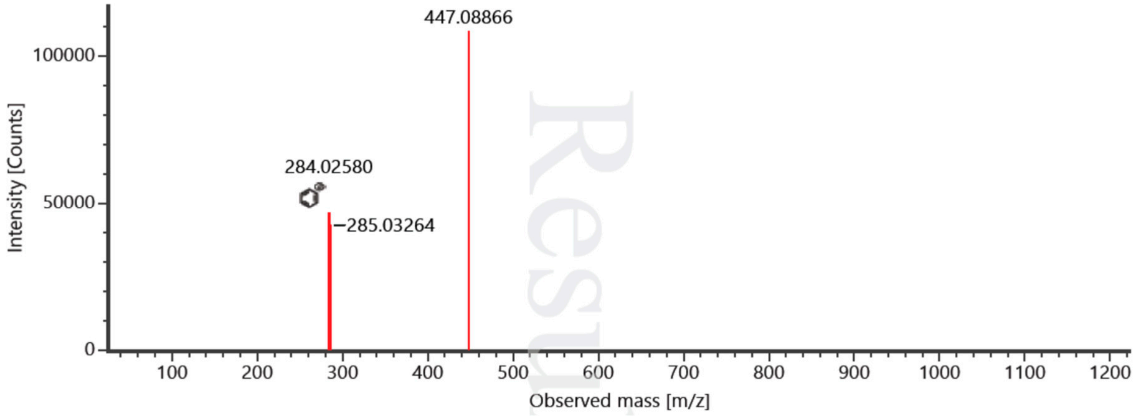

Hydroxygenkwanin

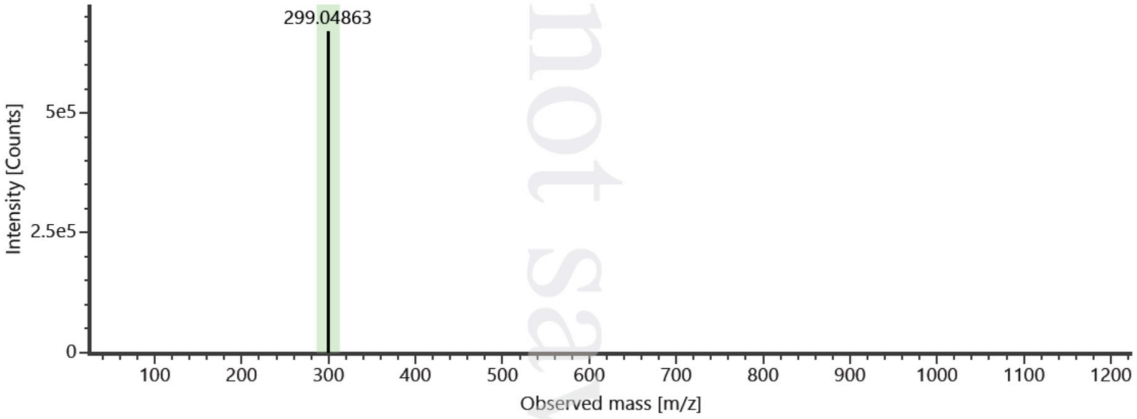

Result not sa

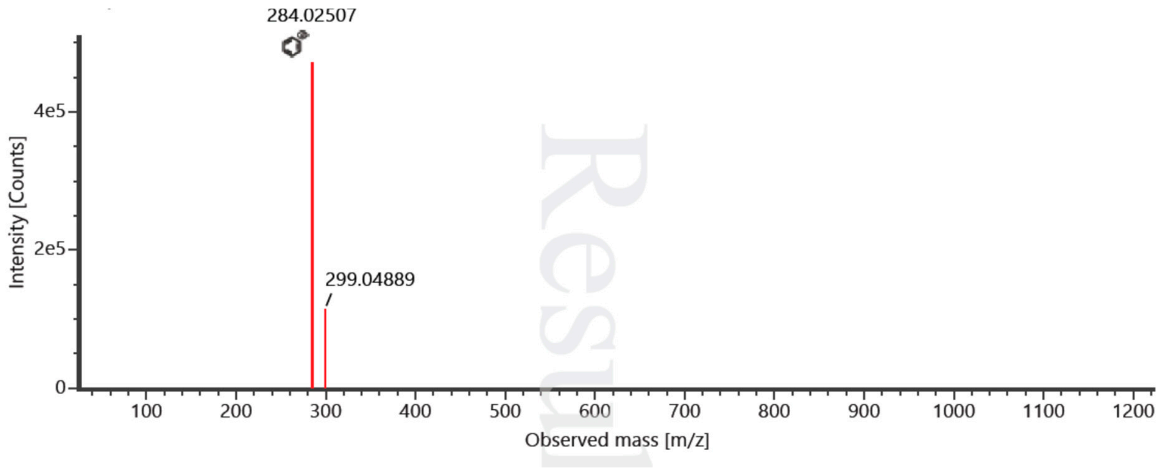
284.02507
299.04889
4e5
2e5
0
Intensity [Counts]
100 200 300 400 500 600 700 800 900 1000 1100 1200
Observed mass [m/z]

Hyperoside

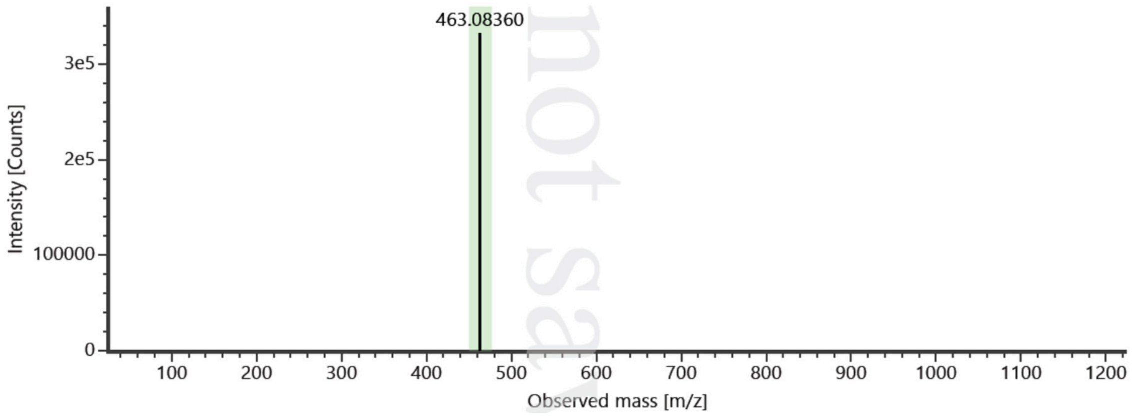
463.08360
3e5
2e5
100000
0
Intensity [Counts]
100 200 300 400 500 600 700 800 900 1000 1100 1200
Observed mass [m/z]

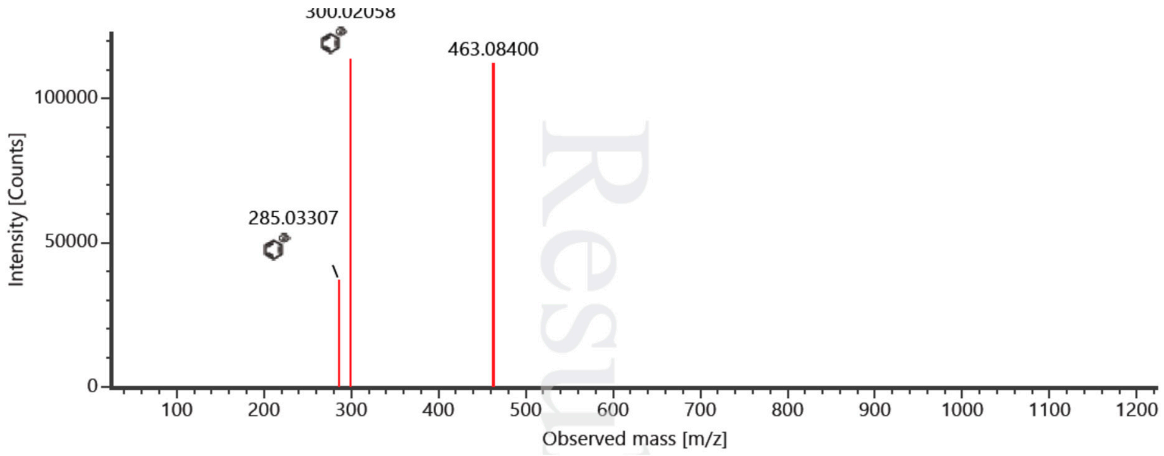

Liquiritin apioside

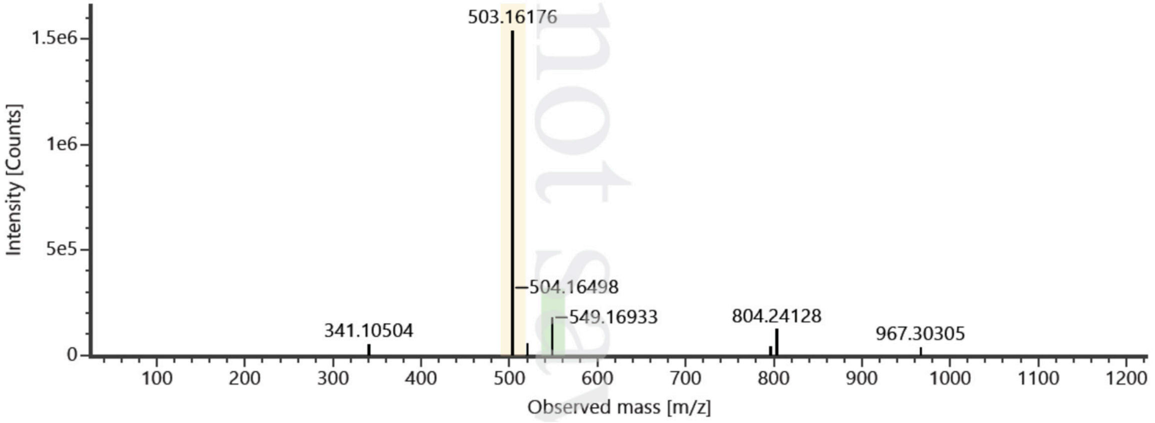

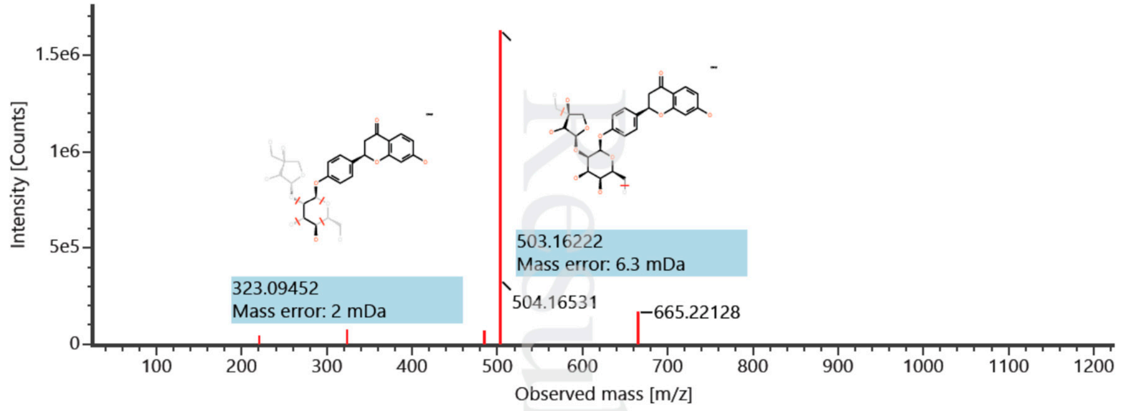

Peonidin-3-O-glucoside

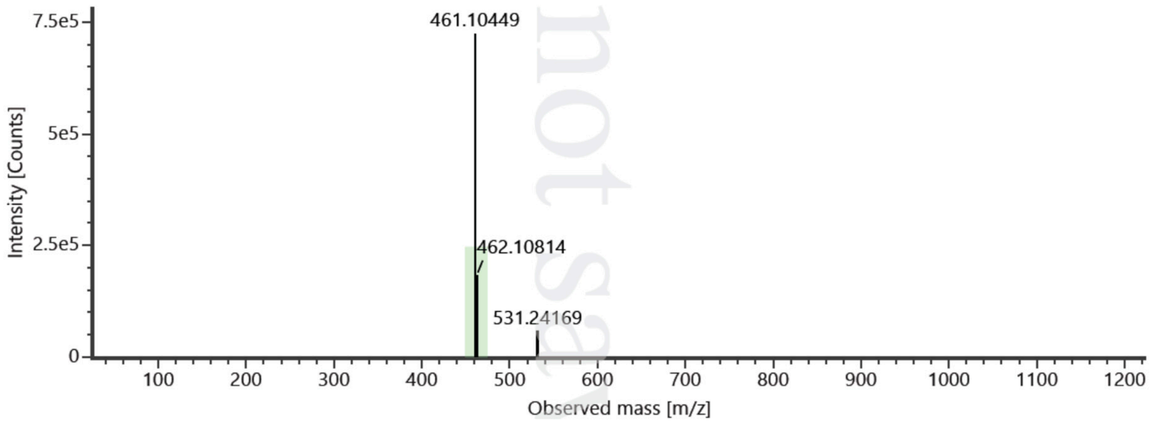

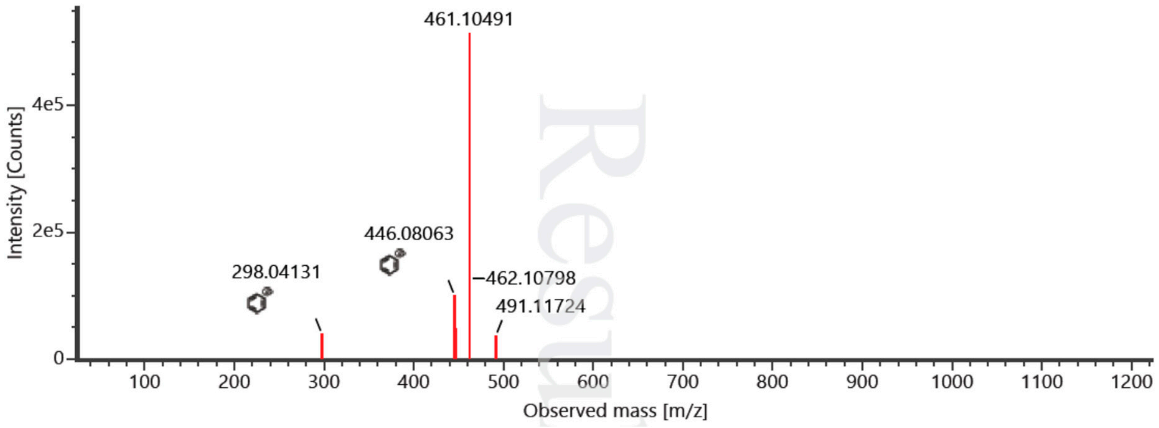

3,4-dicaffeoylquinic acid

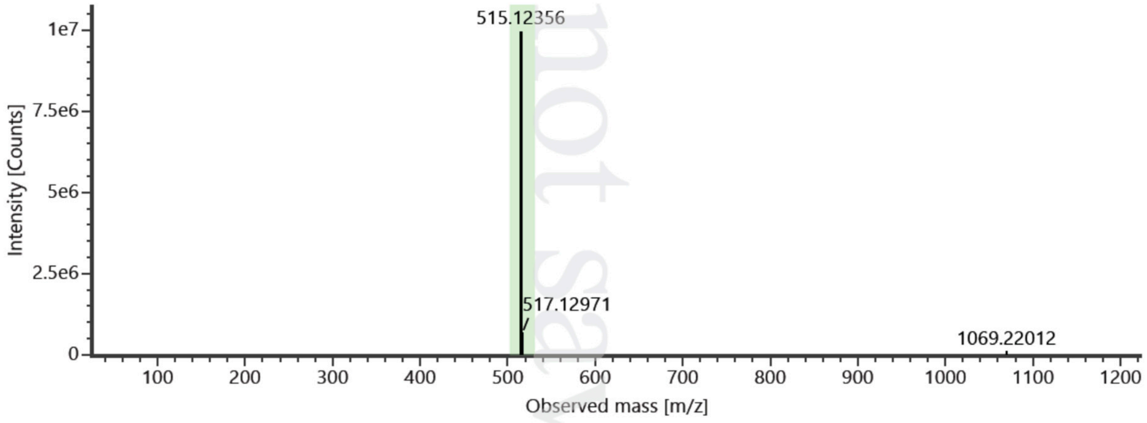

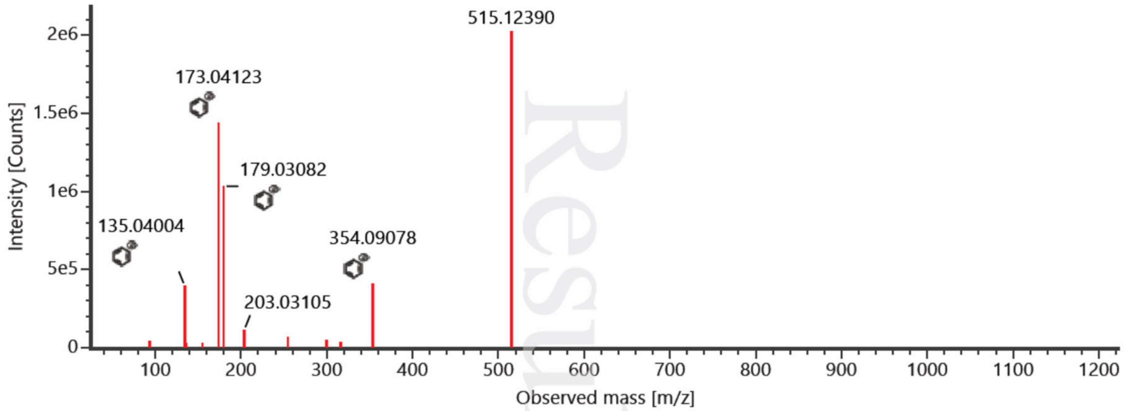
515.12390
173.04123
179.03082
135.04004
354.09078
203.03105
Intensity [Counts]
Observed mass [m/z]

Cyanidin

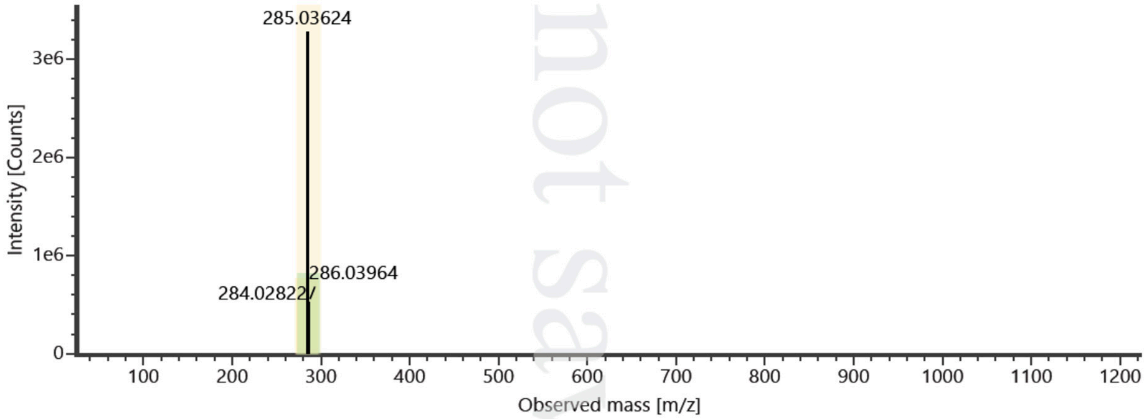
285.03624
286.03964
284.02822
Intensity [Counts]
Observed mass [m/z]

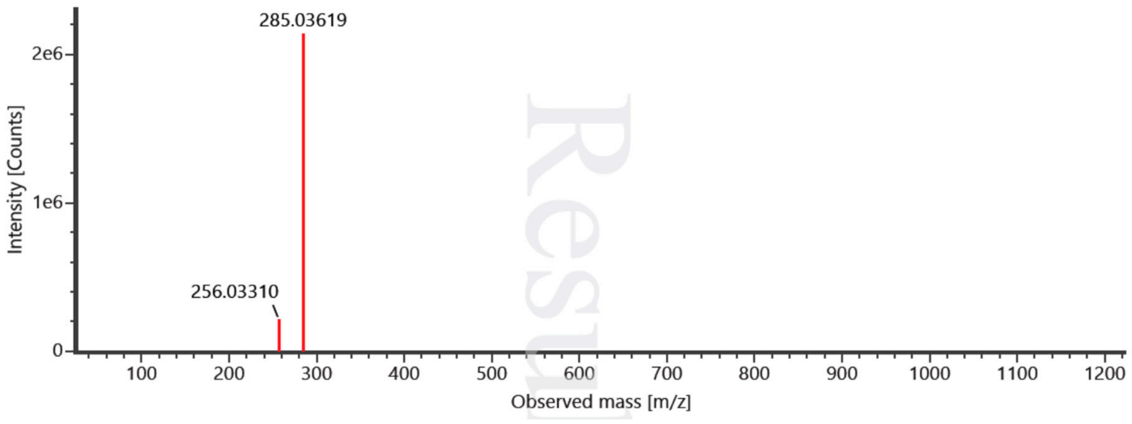
285.03619
2e6
1e6
0
Intensity [Counts]
256.03310
100
200
300
400
500
600
700
800
900
1000
1100
1200
Observed mass [m/z]

Naringin

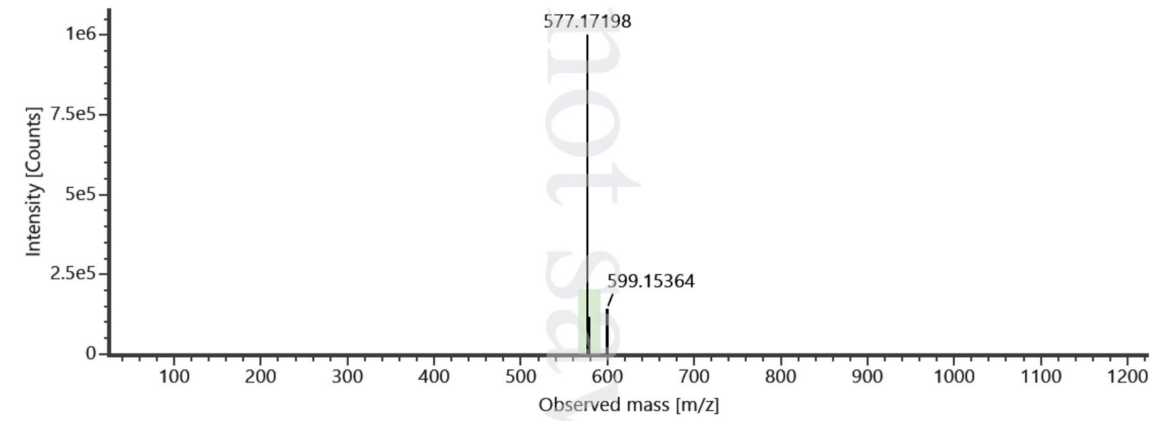

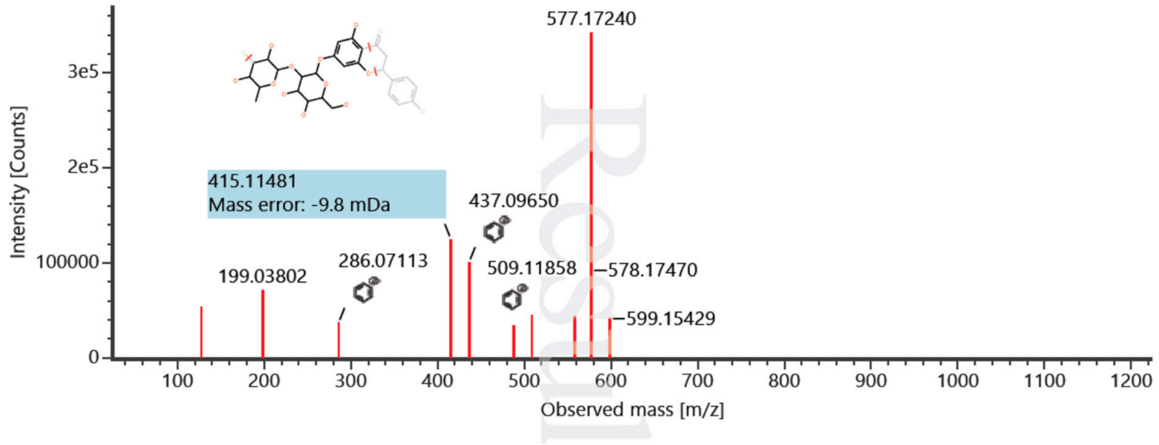

Caffeoyl-6-O-hexoside dihydrate

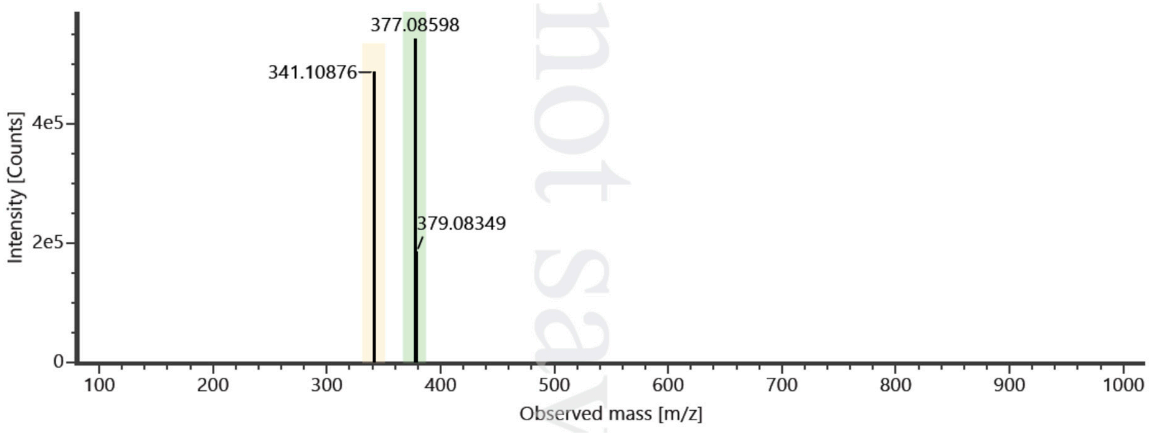

9-hydroxyoctadecadienoic acid

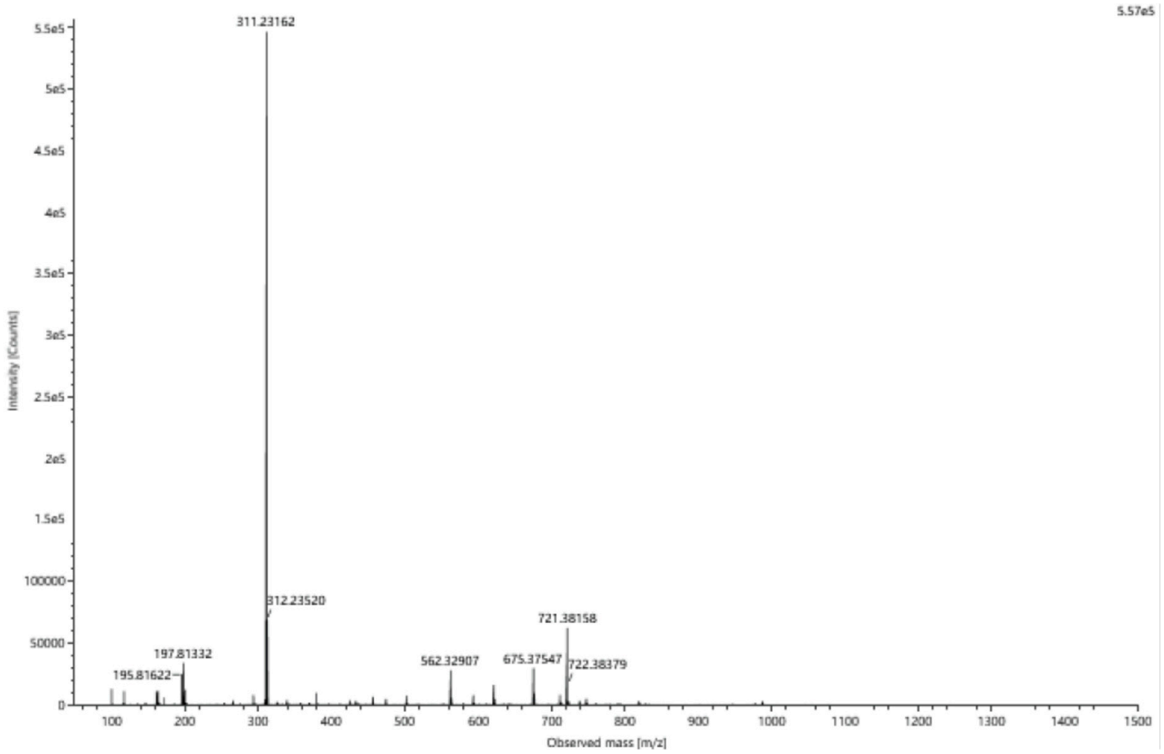
5.57e5
311.23162
312.23520
197.81332
195.81622
562.32907
675.37547
721.38158
722.38379
Intensity [Counts]
Observed mass [m/z]
